# Treatment of sinusitis in children: an Italian intersociety consensus (SIPPS-SIP-SITIP-FIMP-SIAIP-SIMRI-SIM-FIMMG)

**DOI:** 10.1186/s13052-025-01868-1

**Published:** 2025-03-26

**Authors:** Elisabetta Venturini, Margherita Del Bene, Lara Fusani, Eleonora Fusco, Alessia Morlando, Elena Chiappini, Maria Carmen Verga, Giuseppe Di Mauro, Silvia Garazzino, Guido Castelli Gattinara, Susanna Esposito, Eugenia Bruzzese, Nicola Principi, Gian Luigi Marseglia, Fabio Midulla, Mattia Doria, Daniele Donà, Stefania Stefani, Annalisa Capuano, Paolo Biasci, Antonio D’Avino, Annamaria Staiano, Alfredo Guarino, Andrea Lo Vecchio, Luisa Galli

**Affiliations:** 1 Paediatric Infectious Diseases Unit, Meyer Children’s Hospital IRCCS, Florence, Italy; 2 Pediatric Infectious Disease Unit, University Hospital Federico II, Naples, Italy; 3 Department of Health Sciences, University of Florence, Florence, Italy; 4 Department of Clinical and Experimental Medicine, Section of Paediatrics, University of Pisa, Pisa, Italy; 5 Department of Translational Medical Science, Section of Pediatrics, University of Naples Federico II, Naples, Italy; 6 ASL Salerno, Vietri Sul Mare, Salerno, Italy; 7 Pediatric Primary Care, National Pediatric Health Care System, Caserta, Italy; 8 Department of Paediatrics, Infectious Diseases Unit, Regina Margherita Children’s Hospital, University of Turin, Turin, Italy; 9 University Hospital Paediatric Department, Bambino Gesù Children’s Hospital, IRCCS, Rome, Italy; 10 Paediatric Clinic, Department of Medicine and Surgery, University Hospital, University of Parma, Parma, Italy; 11 Emeritus of Pediatrics, University of Milan, Milan, Italy; 12 Department of Pediatrics, Foundation IRCCS Policlinico San Matteo, University of Pavia, Pavia, Italy; 13 Maternal-Science Department, Sapienza University of Rome, Rome, Italy; 14 AULSS 3 Serenissima, Chioggia, Venice Italy; 15 Division of Pediatric Infectious Diseases, Department of Women’s and Children’s Health, University of Padua, Padua, Italy; 16 Department of Biomedical and Biotechnological Sciences, University of Catania, Catania, Italy; 17 Section of Pharmacology ’L. Donatelli’, Department of Experimental Medicine, University of Campania ’Luigi Vanvitelli’, Naples, Italy; 18 Family Pediatrician, Local Health Unit, Livorno, Italy; 19 Federazione Italiana Medici Pediatri (FIMP), Naples, Italy

**Keywords:** Sinusitis, Children, Antibiotics, Treatment

## Abstract

Sinusitis is an inflammation of the mucous membrane of the paranasal sinuses. Bacterial sinusitis usually occurs as a complication of viral infections of the upper respiratory tract and is a frequent cause of medical consultation. The clinical presentation of acute bacterial sinusitis can vary. It most commonly manifests as an upper respiratory tract infection (nasal congestion, postnasal drip, cough) that persists for more than 10 days without clinical improvement. Unfortunately, updated guidelines in paediatric age are not currently available. The purpose of this consensus is to provide guidelines for the therapeutic management of previous healthy paediatric patients with sinusitis. A systematic review was conducted to identify the most recent and relevant evidence. Embase, Scopus, PubMed, and Cochrane databases were systematically screened, combining the terms “children” and “sinusitis” and “antibiotics” and “rhinosinusitis” with a date restriction from 2012 to April 2024, but without language limitations. The certainty of evidence was assessed using the Grading of Recommendations Assessment, Development, and Evaluation (GRADE) methods. The final recommendations were obtained through a Delphi consensus of an expert panel. In children with a diagnosis of uncomplicated acute bacterial sinusitis, made according to strict clinical criteria, systemic empiric antibiotic therapy with amoxicillin or amoxicillin-clavulanic acid is indicated at a high dose (90 mg/kg/day, calculated based on amoxicillin, preferably in 3 daily doses) and for at least 10 days. In children with chronic sinusitis, systemic antibiotic treatment is not recommended, and it is not possible to make any specific recommendation regarding antibiotic agents due to the scarcity of scientific evidence supporting treatment. In conclusion, the diagnosis of sinusitis is primarily clinical, and despite acute sinusitis generally having a favourable course, some cases can present orbital and intracranial complications. The misuse of antibiotics in managing upper respiratory tract infections, including acute sinusitis, and the challenges posed by antibiotic resistance are a current issue in paediatric care. Due to the scarcity, heterogeneity, and poor quality of available evidence either supporting or opposing the use of systemic antibiotic therapy in children with sinusitis prospective studies on larger and more homogeneous cohort are needed.

**Supplementary Information:**

The online version contains supplementary material available at 10.1186/s13052-025-01868-1.

## Background

Sinusitis is an inflammation of the mucous membrane of the paranasal sinuses. Bacterial sinusitis usually occurs as a complication of viral infections of the upper respiratory tract and is a frequent cause of medical consultation. The various paranasal sinuses do not pass through developmental stages simultaneously but have different formation times, with consequent implications in clinical practice [1]. The epidemiology of bacterial sinusitis is not easily defined, and its incidence is currently unknown. Acute sinusitis has a peak incidence between 2 and 6 years of age [2–4], and it is estimated to occur as a complication in 0.5-2% of all upper respiratory tract viral infections [5]. Hoffmans et al. estimate a prevalence of acute bacterial sinusitis of about 18% [1]. Chronic sinusitis is a manifestation of a dysfunction in the interaction mechanisms between the host and environmental factors such as schooling, the presence of siblings, and exposure to passive smoke, and its cumulative prevalence varies widely depending on the studies considered [5–8]. A recent American study, reports a prevalence of chronic sinusitis of 2.1% [9]. In this document, the term “sinusitis” refers to sinusitis of probable bacterial origin (due to the objective difficulties in etiological diagnosis). The definitions reported in Table 1 refers to the duration of clinical manifestation [5, 10–12]. In this document the subacute form is considered together with the acute form.

**Table 1:** Definition of acute, subacute, chronic, recurrent, and recurrent acute sinusitis

Acute sinusitis	1. Sudden onset of ≥ 2 symptoms among nasal obstruction/congestion, nasal discharge, daytime or night-time cough lasting at least 10 days without improvement for a total duration of < 12 weeks/90 days (excluding allergic subjects); 2. Persistent malaise, worsening or new onset of nasal discharge, daytime cough or fever after initial improvement; 3. Severe onset with fever (temperature ≥ 39 °C) and purulent nasal discharge for at least 3 consecutive days.
Subacute sinusitis	Signs/symptoms lasting 4–12 weeks/30–90 days
Chronic sinusitis	Signs/symptoms lasting more than 90 consecutive days
Recurrence	Episode of acute bacterial sinusitis likely lasting less than 30 days, following a previous episode of sinusitis and separated from it by at least 10 days of wellness
Recurrent acute sinusitis	2 episodes of likely acute bacterial sinusitis (at least 3 episodes in 6 months or 4 in 12 months) each lasting less than 30 days.

The majority of studies conducted on aspirates of the paranasal sinuses identify the primary causative agents of sinusitis as *S. pneumoniae*, *H. influenzae*, *M. catarrhalis*, less commonly group A and C streptococci, *Peptostreptococcus spp*, *Eikenella corrodens*, other *Moraxella spp*, *S. aureus*, and anaerobic germs [7, 10, 13–20]. However, recent paediatric data are currently unavailable [6, 21].

Moreover, the role of germs in pediatric chronic sinusitis is controversial, but it is supposed that viral infections cause an alteration of mucus production and mucociliary clearance function increasing the likelihood of bacterial super-infection [22]. The diagnosis of sinusitis is primarily clinical and can vary. It most commonly manifests as an upper respiratory tract infection that persists for more than 10 days without clinical improvement [7]. In some patients, the disease may have a biphasic course: initially, the patient exhibits symptoms of an uncomplicated upper respiratory tract infection that shows clinical improvement after a few days. Thereafter, there is a marked worsening of sinus symptoms, with exacerbation of nasal congestion/drip and/or cough. Fever may also appear or reappear if it had previously resolved. Acute sinusitis may sometimes present as a flu-like syndrome with more severe symptoms from the onset: body temperature > 39 °C with purulent nasal discharge for at least three consecutive days, possibly associated with facial pain and periorbital oedema [2, 7, 10]. In younger children, symptoms are often nonspecific and include irritability, poor appetite, postnasal drip, nasal voice, halitosis and myalgia [23]. School-aged children and adolescents tend to have more specific symptoms such as headache or facial pain. The pain is localized to the cheeks in maxillary sinusitis, to the parietal and temporal regions in posterior ethmoidal sinusitis, above the eyebrows in frontal sinusitis, and in the occipital region in sphenoidal sinusitis. Additionally, facial pain may worsen when the child bends its head forward and can radiate to the dental arch. Halitosis and periorbital oedema may be associated, while laterocervical lymphadenopathy is generally absent [24, 25].

In the case of a single episode of acute sinusitis, microbiological investigations are not indicated, but they may be useful in forms that persist beyond four weeks [26]. Regarding radiological investigations such as X-ray (RX), computed tomography (CT), and magnetic resonance imaging (MRI), they are currently not recommended, especially for differential diagnosis with other upper respiratory tract infections [10].

In 2018, the American College of Radiology suggested the following recommendations:

- Uncomplicated acute sinusitis: imaging is not recommended.
- Persistent, severe, recurrent, or therapy-resistant sinusitis: direct CT is the gold standard.
- Chronic sinusitis: direct CT is not superior to clinical history and examination; therefore, radiological investigations are not necessary [23]. MRI can be useful for studying the mucosa. Standard X-ray presents a high rate of both false positives and false negatives; however, the Waters’ view (occipitofrontal projection) can be useful, offering good specificity and sensitivity compared to nasopharyngeal endoscopy [27]. In cases of therapy-resistant sinusitis, CT with coronal, sagittal, and axial sections is useful and can provide information about complete/partial sinus opacification, the presence of air-fluid levels, mucosal thickness, and any anatomical alterations requiring surgical intervention [28].
- Suspected orbital or intracranial complications: CT of the skull and paranasal sinuses and MRI with contrast are complementary. CT is used to evaluate bone involvement, while MRI is more sensitive for detecting intracranial complications.

Despite acute sinusitis generally having a favourable course, a percentage of cases ranging from 3.7 to 8.8% can present orbital and intracranial complications (as epidural abscess, subdural empyema, parenchymal abscess, meningitis, cerebritis) [10, 29, 30]. In particular, the guidelines of the *National Institute for Health and Care Excellence* (NICE) published in 2017, report a complication rate of 2.5–4.3/1.000.000/year. Other possible complications include osteomyelitis of the maxillary and frontal bones [7]. Complications are more common in children due to the thinner walls of the paranasal sinuses. In particular, children under 5 years of age most commonly experience orbital involvement following ethmoidal sinusitis, while older children have a higher incidence of intracranial complications following frontal sinusitis [31]. Therefore, it is essential to recognize warning signs and symptoms that may indicate a possible complicated sinusitis such as cacosmia, signs of meningitis, altered neurological status and any involvement of the orbital region [32]. Children affected by chronic diseases may have a higher risk of acute sinusitis or may present with recurrent or complicated forms.

The misuse of antibiotics in managing upper respiratory tract infections, including acute sinusitis, and the challenges posed by antibiotic resistance are a current issue in paediatric care. Unfortunately, updated guidelines based on systematic literature review conducted in the last 3–5 years are not currently available. The most recent international guidelines on antibiotic therapy for sinusitis in paediatric patients are the *Infectious Diseases Society of America* (IDSA) guidelines [33], NICE guidelines [34], and practical guidelines of the *American Academy of Pediatrics* (AAP) and *the American Academy of Allergy*, *Asthma*, *and Immunology* [10, 35].

A systematic literature review of the past 10 years was conducted on paediatric sinusitis in otherwise healthy children without comorbidities, and a total of 1,264 articles were identified. Among these, 52 articles were evaluated in full text, including 10 studies for inclusion. Due to the scarcity, heterogeneity, and poor quality of available evidence either supporting or opposing the use of systemic antibiotic therapy in children with sinusitis, recommendations are mostly based on expert panel consensus. It is important to note that these consensus guidelines focus solely on antibiotic therapy and do not address the role of complementary therapies.

For the purpose of this document, treatment failure is defined by the progression or appearance of new signs and symptoms compared to those present at the time of diagnosis or by the lack of clinical improvement in symptoms present at onset within 72 h of therapy [10]. Regarding chronic sinusitis, the response to therapy can be assessed using clinical scores (e.g., Sinonasal Outcome Test, SNOT score) that consider subjective symptoms reported by the patient and objective signs observable during physical examination, as illustrated in Table 2, adapted from EPOS 2020 [5].

**Table 2:** Assessment of clinical control of chronic sinusitis in the previous month (SNOT score)

	Controlled (all the following)	Partially controlled (≥ 1 of the following)	Uncontrolled (≥ 3 of the following)
Nasal congestion	Absent or non-debilitating	Present almost every day	Present almost every day
Rhinorrhea/post-nasal drip	Scant and mucoid	Mucopurulent almost every day	Mucopurulent almost every day
Facial pain/pressure	Absent or non-debilitating	Present almost every day	Present almost every day
Sense of smell	Normal or minimally altered	altered	altered
Fatigue or sleep disturbances	absent	present	present
Rhinoscopy (if available)	Healthy mucosa	Altered mucosa	Altered mucosa
Rescue therapy (in the last 6 months)	Not necessary	Need for 1 cycle	Persistent symptoms despite therapy

## Methods

A systematic review of the literature was conducted to issue recommendations regarding sinusitis treatment. The review was performed according to the GRADE methodology and the PRISMA checklist (Preferred Reporting Items for Systematic Reviews and Meta-analyses) as reported in the Additional files 1–4. The following databases Embase, Scopus, PubMed, and Cochrane were systematically screened with a date restriction from 2012 to April 2024. The MeSH terms “children” and “sinusitis” and “antibiotics” and “rhinosinusitis” were combined as reported in Additional file 1. Randomized controlled trials, observational studies, and systematic reviews with or without meta-analysis on antibiotic therapy in children older than one month with sinusitis were included. Not pertinent studies were excluded. The quality assessment of evidence is provided in Additional File 3 and 4. The following outcomes were considered:

- Effectiveness in reducing the severity and/or duration of symptoms.
- Severe adverse events requiring discontinuation of ongoing antibiotic therapy.
- Development of suppurative and non-suppurative complications.

## Results and recommendations

A summary of the recommendations is shown below. Moreover, an algorithm illustrating the diagnostic and therapeutic approach in children with sinusitis is reported in additional file 5.

### Question 1: is antibiotic treatment indicated in a child with uncomplicated acute sinusitis?

#### Recommendation 1

In children with a diagnosis of uncomplicated acute bacterial sinusitis, made according to strict clinical criteria [represented by: (1) sudden onset of ≥ 2 symptoms among nasal obstruction/congestion, nasal discharge (regardless of its characteristics), daytime or night-time cough lasting at least 10 days without improvement (excluding allergic subjects); or (2) persistent malaise, worsening or new onset of nasal discharge, daytime cough, or fever after initial improvement; or (3) severe onset with fever (temperature ≥ 39 °C) and purulent nasal discharge for at least 3 consecutive days], empiric antibiotic therapy is indicated to achieve rapid symptom improvement (moderate quality of evidence) and reduce the risk of developing potential complications. (Low quality of evidence. Weak recommendation in favour of the intervention)

#### Recommendation 2

In children with subacute sinusitis, it is suggested to prescribe antibiotic therapy if it has not already been administered. (Very low quality of evidence. Expert opinion. Weak recommendation in favour of the intervention)

#### Recommendation 3

According to the principles of “good clinical practice”, in children with subacute sinusitis, if there is no response to antibiotic therapy, it is suggested to seek specialist consultation (paediatrician with expertise in infectious diseases, or otolaryngologist). (Very low quality of evidence. Expert opinion. Weak recommendation in favour of the intervention)

Six articles, evaluating the indication for the use of antibiotic therapy in uncomplicated acute sinusitis in children, were identified. In these studies, the main outcome was the improvement of clinical symptoms, but without a standardized measurement of this outcome. Specifically, two of these are systematic reviews (Cronin et al. [36] and Smith et al. [6]; moderate and low methodological quality, respectively), three randomized controlled trials (RCTs) (Khoshdel et al. [37]; Tugrul et al. [38], and Meltzer et al. [39]; high, moderate, and low methodological quality, respectively), and one retrospective cohort study (Cushen et al. [40]; moderate methodological quality). Additionally, a systematic review (Axiotakis et al. [41]; moderate methodological quality) on the incidence of side effects of antibiotic use in children with acute sinusitis was included. The systematic review and meta-analysis by Cronin et al. [36] includes 4 double-blind RCTs conducted in paediatric populations, enrolling a total of 425 patients, 227 (53.4%) of whom received antibiotic therapy (amoxicillin, amoxicillin-clavulanic acid, or cefuroxime) and 155 (36.5%) were treated with placebo. The clinical outcome was defined as sign/symptom improvement at 10–14 days from the start of therapy [36]. Overall, the meta-analysis shows an advantage in favour of antibiotic therapy use. However, the highlighted methodological limitations raise doubts about its generalizability, therefore, the authors recommend limiting antibiotics prescription to severe cases, since acute sinusitis often resolves spontaneously. The same 4 RCTs were included in the systematic review by Smith et al. [6], in which, due to the heterogeneity of these studies, the authors did not perform a meta-analysis. Specifically, clinical improvement in children receiving the placebo ranged from 14 to 79%, in line with the inclusion criteria of the individual studies. For example, the use of broader inclusion criteria in the study by Kristo et al. [42] might have led to the inclusion of patients with viral upper respiratory infections rather than a confirmed diagnosis of acute sinusitis [42]. The three selected RCTs compared the efficacy of antibiotic therapy versus supportive therapies such as saline nasal irrigation, decongestants, and topical steroids in determining clinical improvement after 14–28 days from treatment start (Meltzer et al. [39]; Khoshdel et al. [37]; Tugrul et al. [38]). All three studies include paediatric patients with uncomplicated acute sinusitis, with persistent symptoms for 7 to 10 days. These characteristics are potentially confounding, possibly suggesting a viral rather than bacterial aetiology and thus affecting the evaluation of the efficacy of antibiotic therapy [7].

In the study by Khoshdel et al. [37], 80 children were randomized into two groups: the intervention group received high-dose amoxicillin (80 mg/kg/day in three doses) for 14 days, saline nasal irrigation (for 5 days), and nasal decongestant (for 2 days); the control group received only nasal irrigation and decongestant therapy. In this study, antibiotic therapy provided limited benefits in the treatment of acute sinusitis. Indeed, after 3 days from the start of therapy, the group that had received amoxicillin reported a significant improvement in symptoms compared to the control group (*p* = 0.001), but after 21–28 days from the start of therapy all patients were cured, and the authors reported no recurrences in both groups [37]. Similar results were reported by Tugrul et al. [38]. Ninety-one children were randomized in two groups comparing amoxicillin-clavulanic acid (90 mg/kg/day in two doses) and nasal decongestant with nasal irrigation and topical steroid. In this study, a faster clinical improvement was recorded in the group of untreated patients (*p* < 0.05 at 7 days), but without significant differences at the end of therapy. The authors therefore propose the use of nasal irrigation combined with topical fluticasone as an alternative therapy in children with acute sinusitis, also in view of the possible side effects that can negatively impact adherence to antibiotic therapy. However, the lack of a placebo group makes these results less generalizable [38]. The effectiveness of topical steroid (mometasone furoate) in reducing symptoms compared to antibiotic therapy with amoxicillin (500 mg three times a day) is also supported by Meltzer et al. based on results obtained from a randomized trial on 981 patients (placebo vs. antibiotic vs. low- or high-dose mometasone furoate) [39]. However, these data are mostly on adults, as only 57 patients aged 12 to 18 years were enrolled, and there was no stratification of results by age group. Additionally, this study is affected by significant publication bias as it was funded by pharmaceutical companies [39].

The incidence of possible side effects secondary to antibiotic intake was evaluated in the systematic review and meta-analysis by Axiotakis et al. [41]. Specifically, the authors analysed the results of five RCTs comparing side effects recorded in patients treated with antibiotics versus placebo. Diarrhoea was the most common side effect, although the risk of developing adverse reactions was not significantly different between the two groups (OR 2.20; 95% CI: 0.86–5.61, I2 = 65). An aspect that can influence the clinician’s judgment and lead to inappropriate antibiotic prescriptions is the fear of possible complications of acute sinusitis. This is a rare event, as reported in 2011 by Hansen et al. [43], which estimates an incidence of suppurative complications in healthy children with acute sinusitis without risk factors of 1/12,000. In this regard, Cushen et al. conducted a retrospective study comparing two large cohorts of children with acute sinusitis treated with antibiotics or placebo [40]. Orbital cellulitis was the only suppurative complication reported, with a cumulative incidence of 4.94 cases per 10,000 episodes of sinusitis (95%CI = 2.89–8.46). In this study, antibiotic therapy significantly reduced the risk of orbital cellulitis compared to the placebo group (OR 0.19; 95%CI = 0.06–0.58). However, given the low incidence of this event in the population, the number of patients needing treatment to prevent one case of orbital cellulitis would be very high (number needed to treat = 691; 95% CI = 260–2695), so the authors advise against routine prescription of antibiotics in children with sinusitis [40]. Indeed, the high percentage of spontaneously resolving acute sinusitis cases in many RCTs seems to be influenced by a less stringent selection of the population, such as to include patients with viral upper respiratory infections. Instead, RCTs like the one conducted by Wald et al. [24], in which the inclusion criteria are very close to the acute sinusitis definition of the guidelines, clearly show the benefit and superiority of antibiotic therapy compared to placebo. International guidelines, therefore, recommend starting empirical antibiotic therapy as soon as a diagnosis of acute bacterial sinusitis is made, or in doubtful cases, considering waiting an additional three days in children with persistent symptoms for at least 10 days without evidence of improvement.

A recent clinical trial involving 515 children aged 2–11 years with a clinical diagnosis of acute sinusitis (excluding those with severe sinusitis) was published. The children were randomized to receive amoxicillin-clavulanate (at a dose of 90 mg/kg of amoxicillin) or placebo for 10 days [44]. At enrolment, all children underwent a nasopharyngeal swab for culture examination to detect *S. pneumoniae*, *H. influenzae*, and *M. catarrhalis*. Both the clinical score and the duration of symptoms were significantly lower in the group treated with amoxicillin-clavulanate compared to the placebo group. However, the efficacy of antibiotic therapy was evident only in children who had detectable bacterial colonization at the nasopharyngeal level, particularly from *S. pneumoniae* or *H. influenzae*. The trial results, while suggesting a possible strategy to reduce the use of antibiotics in acute sinusitis, are difficult to transfer to current clinical practice and confirm the appropriateness of antibiotic prescription in the presence of a clinical diagnosis of acute sinusitis.

Regarding the need for antibiotic therapy in children with symptoms lasting more than 30 days but less than 90 (subacute sinusitis), our literature search identified 3 RCTs, all included in the systematic review by Smith et al. [6]. Of the 3 publications examined, only the one by Dohlman et al. [45] compared systemic antibiotics with placebo. Specifically, the effectiveness of the therapy in reducing patient-reported symptoms and the radiographic appearance of the paranasal sinuses through facial X-rays after 3 and 6 weeks of antibiotic therapy was evaluated. The study compared amoxicillin (30–40 mg/kg/day), amoxicillin-clavulanic acid (30–40 mg/kg/day), and trimethoprim-sulfamethoxazole (8 mg/kg/day calculated on trimethoprim) to placebo. The study found no difference in terms of symptoms or radiographic changes in the group of patients treated with systemic antibiotic therapy compared to placebo. The analysis conducted for each antibiotic also showed no differences in terms of efficacy.

In formulating a recommendation regarding the need for antibiotic therapy, it is important to note that the dose of amoxicillin used in the study (30–40 mg/kg/day) is not currently indicated for the treatment of major respiratory infections, given the poor efficacy of this dosage in the treatment of pneumococcus, which nowadays remains the primary etiological agent.

### Question 2: which first-line topical antibiotic is indicated for uncomplicated acute sinusitis in children?

#### Recommendation 4

In uncomplicated acute sinusitis in children, topical antibiotic therapy should not be prescribed. (Very low quality of evidence. Expert opinion. Strong recommendation against the intervention)

For this question, no studies meeting the inclusion criteria were identified. Studies and systematic reviews are available only concerning the management of chronic sinusitis in adult patients [46]. Even the available guidelines do not mention the use of such therapy in acute sinusitis in paediatric patients [10, 33].

### Question 3: which first-line systemic antibiotic is indicated for uncomplicated acute sinusitis in children?

#### Recommendation 5

In children with uncomplicated acute sinusitis, is suggested an empirical antibiotic therapy with amoxicillin in patients falling under category 1 of the definition (sudden onset of ≥ 2 symptoms among nasal obstruction/congestion, nasal discharge regardless of characteristics, daytime or night-time cough lasting at least 10 days without improvement for a total duration of < 12 weeks/90 days, excluding allergic subjects), and with amoxicillin-clavulanic acid in those falling under category 2 (persistent discomfort, worsening or new onset of nasal discharge, daytime cough, or fever after initial improvement) and 3 of the definition (severe onset with fever (temperature ≥ 39 °C) and purulent nasal discharge for at least 3 consecutive days). (Expert opinion. Weak recommendation in favour of intervention)

#### Recommendation 6

Macrolides and trimethoprim-sulfamethoxazole should not be used in empiric treatment due to high resistance rates of pathogens responsible for acute sinusitis. (Low-quality evidence. Strong recommendation against intervention)

#### Recommendation 7

In children with subacute sinusitis, empirical oral antibiotic therapy with amoxicillin-clavulanic acid is suggested, or tailor antibiotic therapy based on microbiological isolates where available. (Very low-quality evidence. Expert opinion. Weak recommendation in favour of intervention)

Two studies, evaluating first-line systemic antibiotic therapy in children with acute sinusitis, were identified. One of these is a systematic review (Smith et al. [6]; low methodological quality), and the other one is a RCT (Lari et al. [47]; very low methodological quality). Smith et al. systematic review included five RCTs conducted in the paediatric population, comparing different treatment regimens such as amoxicillin, amoxicillin-clavulanate, macrolides, cephalosporins, and sulphonamides [6]. Specifically, two RCTs compared amoxicillin or amoxicillin-clavulanate with cephalosporins, evaluating clinical improvement after 10–14 days of therapy (Poachanukoon et al. [48]; Wald et al. [49]). In the study by Wald et al., the cure rate in the amoxicillin group was similar to that one in the cephalosporin group (81% vs. 78%, respectively) [49]. Poachanukoon et al. also reported a comparable recurrence risk in the amoxicillin-clavulanate group compared to those who received cephalosporins (11.1% vs. 9.1%, *p* = 0.78) [48]. A third RCT, also included in Smith’s review [6], compared the efficacy of macrolides to a third-generation cephalosporin, analysing clinical outcomes (resolution of symptoms) after 14–20 days of treatment [50]. No meta-analysis was conducted due to significant study heterogeneity, methodological differences and the use of various molecules and dosages. However, none of these studies demonstrated a clear advantage of one treatment regimen over another, with a cure rate of approximately 80% across the studies. An important aspect affecting the applicability of these results is the lack of a placebo group in each of the selected studies, which could not exclude the possibility that many children may improve independently of the prescribed therapy. Furthermore, adverse effects related to antibiotic therapy were reported in all included studies. Specifically, Poachanukoon et al. reported a significantly higher incidence of diarrhoea in patients treated with amoxicillin-clavulanate compared to cephalosporins (18.1% vs. 4.5%; *p* = 0.02) [48]. In all cases, diarrhoea was self-limiting without the need for further treatment (Smith et al., [6]; Poachanukoon et al. [48]). These findings are consistent with those reported in Axiotakis’ review, where amoxicillin (8.7%, 95% CI: 0.0-28.1; I2 = 89) and amoxicillin-clavulanate (13.7%, 95% CI: 6.1–23.4; I2 = 73) were associated with a higher incidence of adverse reactions compared to cephalosporins (5.3%, 95% CI: 1.5–10.9%, I2 = 49) [41].

Lari et al. [47] randomized 99 patients, aged over 12 years, into two groups. Among these, 57 were treated with cefuroxime and 42 with amoxicillin-clavulanic acid, finding no significant differences in clinical outcomes between the two groups. However, the results of this study cannot be generalized to the paediatric population because the number of patients under 18 years old is not reported, and there was no age stratification of the outcomes. All the available International guidelines recommend β-lactams as the first-line antibiotics for children with acute sinusitis. The IDSA guidelines [33], for instance, advise against using macrolides or trimethoprim-sulfamethoxazole due to high resistance rates among strains of *S. pneumoniae* and *H. influenzae*. Specifically, the IDSA guidelines recommend amoxicillin-clavulanic acid over amoxicillin due to the increasing prevalence of β-lactamase-producing strains of *H. influenzae* and *M. catarrhalis* [33]. This increase is partly due to the widespread use of pneumococcal vaccination, which has reduced the incidence of this pathogen [33]. Literature reports 10–42% of *H. influenzae* and nearly 100% of *M. catarrhalis* isolates are β-lactamase-positive [51]. Similarly, NICE guidelines also recommend amoxicillin-clavulanic acid as the first-line therapy. AAP guidelines are less categorical about choosing between amoxicillin and amoxicillin-clavulanic acid but suggest amoxicillin-clavulanic acid in cases where there is a risk of resistance to amoxicillin. This includes children with moderate to severe sinusitis, those under 2 years old, those attending day-care, or those who have recently received antibiotic therapy [10]. Regarding patients with subacute sinusitis, three RCTs were selected, all included in Smith et al.‘s systematic review [6]. Specifically, Dohlman et al. compared the efficacy of three antibiotic molecules (amoxicillin, amoxicillin-clavulanic acid, or trimethoprim-sulfamethoxazole) to placebo [45]. Moreover, Ng et al. compared a 15-day course of amoxicillin-clavulanic acid with a 3-day course of azithromycin [52], while El-Hennawi et al. evaluated empirical antibiotic therapy with amoxicillin-clavulanic acid versus targeted therapy based on nasal mucous culture antibiogram results in patients under 2 years old [53]. In particular, targeted antibiotic demonstrates a lower failure rate compared to empirical therapy with amoxicillin-clavulanic acid for 14 days (15% vs. 35%), with significant improvement in nasal obstruction (*p* = 0.037) and nasal secretions (*p* = 0.003) [53]. It is noteworthy that in the subgroup of patients receiving targeted therapy with amoxicillin-clavulanic acid at a dose of 90 mg/kg/day, all patients (*n* = 6) showed marked improvement in nasal symptoms at the end of treatment.

### Question 4: what dosage of first-line systemic antibiotic therapy is recommended for children with uncomplicated acute sinusitis?

#### Recommendation 8

For the treatment of uncomplicated acute sinusitis, considering the resistance rate of *S. pneumoniae* strains in Italy, which is consistently ≥ 10%, amoxicillin or amoxicillin-clavulanic acid should be prescribed at a high dose (90 mg/kg/day, calculated based on amoxicillin, preferably in 3 daily doses). (Very low-quality evidence. Expert opinion. Weak recommendation in favour of the intervention)

For this question, no studies meeting the inclusion criteria were identified.

The international IDSA guidelines recommend using a high dose of amoxicillin-clavulanic acid (90 mg/kg/day in 2 doses, calculated based on amoxicillin) in cases from areas with high rates of penicillin-resistant *S. pneumoniae* strains (≥ 10%), severe infection (body temperature at least 39 °C and/or suppurative complications), age less than 2 years (due to higher risk of invasive complications from capsulated bacteria), community frequency, recent hospitalization, use of antibiotics in the previous month, and immunosuppression. This recommendation is weak and supported by moderate-quality evidence [33]. According to data from the latest report of the national antibiotic resistance surveillance (AR-ISS 2022), penicillin-resistant *S. pneumoniae* strains are at 12.8%; these data align with those reported by the European Centre for Disease Prevention and Control (ECDC), which indicate a rate of 10–25%. The high dose of amoxicillin-clavulanic acid was also recommended in a recent narrative review in patients at risk of severe sinusitis or possible antibiotic resistance [25]. In other situations, according to AAP guidelines, the standard dose of amoxicillin (45 mg/kg/day in 2 doses), with or without clavulanic acid, is recommended.

A recent RCT conducted in adults indicated no significant differences in clinical improvement after 3 days of treatment using high-dose amoxicillin-clavulanic acid compared to the standard dose, leading to the study’s termination [54]. However, it is important to note that the study population did not include patients with risk factors for severe forms (exclusion criteria included immune system deficiencies, high risk of amoxicillin-resistant bacteria, severe infection, and antibiotic therapies in the previous 3 months for past episodes of sinusitis).

### Question 5: which is the duration of oral antibiotic therapy in children with uncomplicated acute sinusitis?

#### Recommendation 9

In children with uncomplicated acute sinusitis, oral antibiotic therapy should be continued for at least 10 days. (Low quality of evidence. Expert opinion. Strong recommendation in favour of the intervention)

For this question, no studies meeting the inclusion criteria were identified.

The IDSA international guidelines recommend an antibiotic course of 10–14 days, although this recommendation is considered weak and supported by moderate-low quality evidence [33].

The AAP guidelines, on the other hand, recommend to continue therapy for 7 days after clinical improvement [10]. In the review by DeMuri et al., a therapy duration of 10 days is suggested in case of a rapid response to antibiotic therapy; otherwise, it is recommended to continue therapy until seven days after the complete clinical resolution, as also indicated by the AAP [7, 10]. Another recent narrative review suggests a therapy duration of 5 days [55]. Another review by Dawson-Hahn et al., drawing data from a 2009 systematic review by Falagas et al., which included exclusively 4430 adult patients, shows that there was no significant difference in the clinical course of patients treated for 3–7 days or 6–10 days, but only a lower risk of side effects in the group with shorter duration (OR: 0.79, 95% CI: 0.63–0.89) [56, 57].

### Question 6: which is the recommended second-line systemic antibiotic therapy for children with acute sinusitis?

#### Recommendation 10

In children with acute sinusitis who are on first-line empirical antibiotic therapy, if there is clinical worsening (or no improvement after 48–72 h), a switch to second-line antibiotic therapy should be considered. (Low quality of evidence. Strong recommendation in favour of the intervention)

#### Recommendation 11

In children who not respond to first-line therapy with amoxicillin, a second-line antibiotic therapy with amoxicillin-clavulanic acid is suggested. For children with a poor response to first-line therapy with amoxicillin-clavulanic acid, a second-line therapy with a combination of a second or third-generation cephalosporin (e.g., cefixime, cefpodoxime, cefuroxime) and clindamycin is suggested. (Low quality of evidence. Weak recommendation in favour of the intervention).

For this question, no studies meeting the inclusion criteria were identified.

The IDSA international guidelines recommend considering an alternative antibiotic therapy in case of clinical worsening after 48–72 h or lack of improvement after 3–5 days of therapy [33].

The AAP guidelines suggest, based on evidence from RCTs, to reassess the patient after 72 h from the start of therapy before changing the antibiotic regimen [10]. According to the AAP, in case of clinical worsening or lack of improvement during therapy with amoxicillin, it is recommended to switch to high-dose amoxicillin-clavulanic acid [10]. In case of clinical worsening or lack of improvement during therapy with high-dose amoxicillin-clavulanic acid, both the IDSA and AAP guidelines recommend switching to a combination therapy with clindamycin (30–40 mg/kg/day in 3–4 doses) and second- or third-generation cephalosporin (e.g., cefixime), or linezolid (in areas with high levels of *S. pneumoniae* resistant to clindamycin) and cefixime. A 2013 narrative review also emphasizes the difficulty in choosing a second-line agent for sinusitis [7]; cephalosporins such as cefpodoxime (10 mg/kg/day in 2 daily doses) or cefuroxime axetil (20–30 mg/kg/day in 2 daily doses) may be used. The three main pathogens (*S. pneumoniae*, *H. influenzae*, and *M. catarrhalis*) remain susceptible in 95–100% of cases to ceftriaxone. The susceptibility of *S. pneumoniae* to cefdinir, cefpodoxime, and cefuroxime varies from 60 to 75%, and that of *H. influenzae* from 85 to 100% [10]. Levofloxacin (16 mg/kg/day in 2 daily doses) is also effective in such situations, but its use is justified only in the absence of alternatives due to side effects and lack of authorization for paediatric use.

### Question 7: is systemic antibiotic treatment indicated in children with chronic sinusitis?

#### Recommendation 12

In children with chronic sinusitis, systemic antibiotic treatment is not recommended. (Quality of evidence very low. Weak recommendation against the intervention)

#### Recommendation 13

Before perform invasive diagnostic interventions, including radiological procedures, it is suggested to prescribe a course of antibiotic therapy if not previously administered. (Quality of evidence very low. Expert opinion. Weak recommendation in favour of the intervention)

#### Recommendation 14

In cases of chronic sinusitis, before undertaking invasive diagnostic interventions, including radiological procedures, according to the principles of good clinical practice, a specialist consultation (paediatric infectious disease specialist, otolaryngologist) is recommended. (Quality of evidence very low. Expert opinion. Weak recommendation in favour of the intervention)

The systematic literature review on chronic sinusitis (persistent symptoms > 90 days) identified 1264 studies, of which only 2 met the inclusion criteria defined in the methodology (a systematic review and an observational study).

The systematic review by Head et al. included 5 RCTs (*n* = 293) testing various antibiotic treatments [58]. Any clinical or radiological benefit for patients treated with systemic antibiotic therapy was found if compared to placebo or anti-inflammatory therapy. However, despite the document is of high quality, of the 5 studies included only one RCT enrolled children, making the conclusions less generalizable to the paediatric age group. In the 4 RCTs involving adult patients, the use of different antibiotic agents such as macrolides, tetracyclines, and cephalosporins did not show any advantage in terms of clinical response to therapy (SNOT score at 3 and 6 months, see Table 2), patient-reported symptoms, or side effects in individuals with chronic sinusitis. In the single RCT carried out in paediatric patients, Otten et al. evaluated the efficacy of therapy with Cefaclor (20 mg/kg/day for 7 days) vs. placebo in reducing sinusitis symptoms reported by parents and resolving radiographic evidence of sinus opacity in 75 children (37 intervention + 38 placebo) with a mean age of 5 years (range 2–12 years) [59]. At 6 weeks of therapy, 64.8% of patients allocated to antibiotic therapy and 52.5% of those who received placebo were reported as cured (*p* = 0.28). However, it should be noted that at 6 weeks, 50% of patients with bilateral sinus opacity and 66% of patients with less severe presentations experienced spontaneous recovery, independent of antibiotic treatment. Although a further follow-up visit was planned, the authors did not report the results observed 12 weeks after the end of antibiotic therapy. However, the small sample size, short treatment duration, and clinical outcome considered do not allow to conclusive results [59].

The second study is a retrospective observational study conducted on only 6 children (age < 15 years) with chronic sinusitis resistant to anti-inflammatory/symptomatic therapy, undergoing antibiotic therapy with low-dose macrolides [60]. Besides referring to a population already unsuccessfully treated with non-antibiotic first-line therapy, the study was imprecise and poorly reproducible. The authors did not report the specific molecules administered to each patient, nor did they accurately describe the duration, dose, timing of exposure to treatment, nor accurately reporting the methods for evaluating the outcome. The extremely low methodological quality of the article does not provide evidence to answer the question.

### Question 8: which is the first-line systemic antibiotic therapy indicated in children with chronic sinusitis?

#### Recommendation 15

In children with chronic sinusitis, it is not possible to make any specific recommendation regarding antibiotic agents due to the scarcity of scientific evidence supporting treatment. (Quality of evidence very low. Expert opinion. Weak recommendation in favour of the intervention)

#### Recommendation 16

In children for whom antibiotic treatment has been decided based on the reasons described in recommendation 24, it is suggested to prescribe systemic antibiotic therapy with amoxicillin-clavulanate, if not previously administered. (Quality of evidence very low. Expert opinion. Weak recommendation in favour of the intervention)

Considering the poor quality of the studies identified in the literature search, the small sample sizes, and the clinically marginal and poorly reproducible results, it is not possible to recommend the use of a specific antibiotic agent for the treatment of children with chronic sinusitis. Although the data from the study by Otten et al. are undoubtedly affected by serious methodological biases, therapy with cefaclor at a daily dose of 20 mg/kg for 7 days showed no efficacy in reducing nasal symptoms reported by caregivers compared to placebo [59].

### Question 9: what is the duration of systemic antibiotic therapy in children with chronic sinusitis?

#### Recommendation 17

In the absence of scientific evidence, it is not possible to formulate any specific recommendation. In children with chronic sinusitis, the duration of any systemic antibiotic therapy should be evaluated in consultation with a specialist (paediatric infectious disease specialist, otolaryngologist), taking into account previous antibiotic therapies, resistance patterns, and host-related risk factors. (Low-quality evidence. Expert opinion. Weak recommendation in favour of the intervention)

According to the systematic literature review, no clinical studies comparing different therapeutic regimens in the treatment of chronic sinusitis in paediatric patients have emerged.

### Question 10: which is the recommended treatment for a patient with uncomplicated sinusitis and penicillin allergy?

#### Recommendation 18

In patients diagnosed with sinusitis suspected of amoxicillin allergy and with low risk of severe allergic reaction, it is recommended to consider a third-generation cephalosporin (e.g., cefixime, cefpodoxime, ceftibuten) as an alternative therapy for at least 10 days. Macrolides should be avoided due to their limited activity against the likely pathogens involved. The prescription of a quinolone should be limited to patients classified under category 3 of the acute sinusitis definition and with a concurrent high risk of allergic reaction. This prescription should be discussed with a paediatric infectious disease specialist. (Quality of evidence very low, Expert opinion. Weak recommendation in favour of the intervention).

Currently, there are no studies of adequate scientific quality addressing specifically the treatment of uncomplicated sinusitis in patients allergic to penicillin.

NICE guidelines recommend the use of macrolides (such as clarithromycin) or doxycycline (> 12 years old) in patients allergic to penicillin. However, this recommendation may not be universally applicable due to the epidemiology of bacterial resistance. In Italy, for instance, resistance rates among *S. pneumoniae* and *H. influenzae* to macrolides preclude their use as first-line drugs in patients allergic to penicillin, due to the high risk of therapeutic failure.

Some authors and older guidelines (IDSA and AAP 2013) differentiate treatment according to the severity of allergy: for non-severe allergy to amoxicillin, the use of an oral third-generation cephalosporin is suggested, while severe allergy prompts the use of levofloxacin [10, 33]. The consensus by Orlandi et al. 2016 cites IDSA and AAP guidelines [61]. The systematic review by Smith et al. mentions in a table these guidelines: in cases of allergies, the Cincinnati Children’s Hospital guideline (2006) indicates second-line drugs (cefuroxime, cefpodoxime, and cefdinir for non-type I allergic reactions) [6]; the SAHP 2004 guideline (Sinus and Allergy Health Partnership) proposes cotrimoxazole or macrolides for mild sinusitis, and the same or clindamycin for moderate sinusitis or in cases of prior antibiotic therapy [62].

### Question 11: which antibiotic therapy is recommended for a child with a recurrence of uncomplicated acute sinusitis?

#### Recommendation 19

In case of recurrence of uncomplicated acute sinusitis without risk factors, it is suggested to use antibiotic therapy with amoxicillin-clavulanate if the previous episode was treated with amoxicillin. If the previous therapy with amoxicillin-clavulanate had poor compliance, it is recommended to repeat the therapeutic cycle with the same antibiotic while ensuring adherence to treatment. For patients who have already been treated with amoxicillin-clavulanate with good compliance, switching to second-line antibiotic therapy is suggested. The panel also recommends, in accordance with good clinical practice principles, seeking specialist consultation (paediatric infectious diseases specialist, otolaryngology). (Quality of evidence very low. Expert opinion. Weak recommendation in favour of the intervention)

There are no studies of adequate scientific quality available in the literature specifically addressing the treatment of recurrent uncomplicated acute sinusitis. Some authors suggest using the same antibiotics used in the first episode of acute sinusitis for recurrences, as the microbiology overlaps in children without risk factors [10, 63].

### Question 12: is antibiotic prophylaxis recommended in children with recurrent sinusitis?

#### Recommendation 20

The available literature is not sufficient to formulate a specific recommendation on antibiotic prophylaxis in children with recurrent sinusitis. (Low-quality evidence. Expert opinion. Weak recommendation in favour of the intervention)

Recurrent acute bacterial sinusitis (RARS) is rare in healthy children. Therefore, in case of recurrence (episodes of bacterial sinus infection lasting < 30 days and separated by intervals of wellness of at least 10 days – at least 3 episodes in 6 months or 4 in 12 months), it is necessary to investigate the presence of allergies (especially allergic rhinitis), non-allergic rhinitis, immune deficiencies (e.g., IgG subclass deficiency or IgA deficiency), cystic fibrosis, gastroesophageal reflux, ciliary motility disorders, obstructive anatomical abnormalities (e.g., adenoid hypertrophy).

The efficacy of antibiotic prophylaxis has not been systematically evaluated so far. The systematic literature review identified only one prospective double-blind randomized controlled study. In this study the efficacy of azithromycin prophylaxis (5 mg/kg/day three times a week for 12 months) was compared to placebo in 20 children (+ 20 controls) aged 5 to 15 years with RARS but without allergic rhinitis. Prophylaxis was statistically effective in reducing the number of episodes per year (from 5 to 0.5) and the severity of symptoms. No side effects were reported in either the treatment or control group. The study did not evaluate the impact of prophylaxis on symptom duration or the risk of suppurative/non-suppurative complications. Moreover, approximately 80% of children included in the study (in both groups) had a subclass IgG deficiency [64]. No RCT has evaluated other molecules as prophylaxis. A retrospective cohort study from 2015 by Veskitkul et al., analysed data of 94 children with RARS [63]. Among these, 40 (61.5%) received prophylactic treatment with amoxicillin or azithromycin: 32 patients (80%) responded to prophylaxis (= 50% reduction in sinusitis episodes in the 12 months following initiation of prophylaxis).

Lastly, a systematic review by Smith et al. cites a randomized study from 1993 by Gandhi A et al. on antibacterial prophylaxis in children with chronic sinusitis: 26 out of 86 children received antibiotic prophylaxis (not specified further) for 12 months, and of these 26, 73% (*n* = 19) achieved a 50% reduction in episodes [6, 65].

## Conclusion

The epidemiology of bacterial sinusitis is not easily defined, and its incidence is currently unknown. Acute sinusitis has a peak incidence between 2 and 6 years of age [2–4], and it is estimated to occur as a complication in 0.5-2% of all upper respiratory tract viral infections [5]. Despite acute sinusitis generally having a favourable course, a percentage of cases ranging from 3.7 to 8.8% can present orbital and intracranial complications [10, 29]. Complications are more common in young children due to the thinner walls of the paranasal sinuses. Although rare, these conditions can lead to neurological sequelae (including blindness), and even death [10]. The misuse of antibiotics in managing upper respiratory tract infections, including acute sinusitis, and the challenges posed by antibiotic resistance are a current issue in paediatric care. Unfortunately, updated guidelines based on systematic literature review conducted in the last 3–5 years are not currently available.

In children with a diagnosis of uncomplicated acute bacterial sinusitis, made according to strict clinical criteria, empiric antibiotic therapy for at least 10 days is indicated to achieve rapid symptom improvement. Amoxicillin is suggested in patients with sudden onset of ≥ 2 symptoms among nasal obstruction/congestion, nasal discharge regardless of characteristics, daytime or night-time cough lasting at least 10 days. Amoxicillin-clavulanic acid should be the choice for children with persistent discomfort, worsening or new onset of nasal discharge, daytime cough, or fever after initial improvement and for those with severe sinusitis. Amoxicillin or amoxicillin-clavulanic acid should be prescribed at a high dose (90 mg/kg/day, calculated based on amoxicillin, preferably in 3 daily doses). For children with a poor response to first-line therapy with amoxicillin-clavulanic acid, a second-line therapy with a combination of a second or third-generation cephalosporin (e.g., cefixime, cefpodoxime, cefuroxime) and clindamycin is suggested. Macrolides and trimethoprim-sulfamethoxazole should not be used in empiric treatment due to high resistance rates of pathogens responsible for acute sinusitis.

According to the systematic literature review, no clinical studies comparing different therapeutic regimens in the treatment of chronic sinusitis in paediatric patients have emerged. In the absence of scientific evidence, it is not possible to formulate any specific recommendation. In children with chronic sinusitis, the drug of choice and treatment duration should be evaluated in consultation with a paediatric infectious disease specialist, taking into account previous antibiotic therapies, resistance patterns, and host-related risk factors.

Due to the scarcity, heterogeneity, and poor quality of available evidence either supporting or opposing the use of systemic antibiotic therapy in children with sinusitis prospective studies on larger and more homogeneous cohort are needed.

## Electronic supplementary material

Below is the link to the electronic supplementary material.

Supplementary Material 1

Supplementary Material 2

Supplementary Material 3

Supplementary Material 4

Supplementary Material 5

## Data Availability

The datasets supporting the conclusions of this article are included within the article (and its additional files).

## Abbreviations

RX: X-ray
CT: Computed tomography
MRI: Magnetic resonance imaging
CI: Confidence interval
OR: Odds ratio
SR: Systematic review
RCT: Randomized controlled trial
RARS: Recurrent acute bacterial sinusitis
