# Supplementary material for: Treatment of sinusitis in children: an Italian intersociety consensus (SIPPS-SIP-SITIP-FIMP-SIAIP-SIMRI-SIM-FIMMG)
Source: Ital J Pediatr. 2025 Mar 26;51:102. doi: 10.1186/s13052-025-01868-1 (PMC11948864; doi:10.1186/s13052-025-01868-1)
Supplement: Supplementary file 1 — Supplementary Material 1 [file 13052_2025_1868_MOESM1_ESM.docx]

**PICOs**

1. **Is antibiotic treatment indicated in a child with uncomplicated acute sinusitis?**

P In children with acute sinusitis without specific risk factors

I1 topical antibiotic therapy

I2 systemic antibiotic therapy

C compared to anti-inflammatory therapy alone

O1 is equally effective in reducing the severity and/or duration of symptoms?

O2 does it modify the risk of recurrences?

O3 does it modify the risk of suppurative complications (orbital cellulitis, brain abscess, others)?

O4 does it modify the risk of non-suppurative complications (cavernous sinus thrombosis, others)?

O5 does it modify the risk of side effects?

1. **Which first-line topical antibiotic is indicated for uncomplicated acute sinusitis in children?**P In children with acute sinusitis without specific risk factors

I1 topical antibiotic therapy with aminoglycosides

I2 topical antibiotic therapy with fluoroquinolones

C compared to topical antibiotic therapy with cephalosporins

O1 is equally effective in reducing the severity and/or duration of symptoms?

O2 does it modify the risk of recurrence?

O3 does it modify the risk of suppurative complications (orbital cellulitis, brain abscess, others)?

O4 does it modify the risk of non-suppurative complications (cavernous sinus thrombosis, others)?

O5 does it modify the risk of side effects?

1. **Which first-line systemic antibiotic is indicated for uncomplicated acute sinusitis in children?**

   P In children with acute sinusitis without specific risk factors

I1 antibiotic therapy with amoxicillin

I2 antibiotic therapy with macrolides

I3 antibiotic therapy with cephalosporins

I4 antibiotic therapy with fluoroquinolones

C compared to antibiotic therapy with amoxicillin-clavulanate

O1 is equally effective in reducing the severity and/or duration of symptoms?

O2 does it modify the risk of recurrence?

O3 does it modify the risk of suppurative complications (orbital cellulitis, brain abscess, others)?

O4 does it modify the risk of non-suppurative complications (cavernous sinus thrombosis, others)?

O5 does it modify the risk of side effects?

1. **What dosage of first-line systemic antibiotic therapy is recommended for children with uncomplicated acute sinusitis?**

P In children with acute sinusitis without specific risk factors

I low-dose amoxicillin-clavulanate (50 mg/kg/day) in 2-3 daily doses

C compared to high-dose amoxicillin-clavulanate (90 mg/kg/day) in 2-3 daily doses

O1 is equally effective in reducing the severity and/or duration of symptoms?

O2 does it modify the risk of recurrence?

O3 does it modify the risk of suppurative complications (orbital cellulitis, brain abscess, others)?

O4 does it modify the risk of non-suppurative complications (cavernous sinus thrombosis, others)?

O5 does it modify the risk of side effects?

1. **Which is the duration of oral antibiotic therapy in children with uncomplicated acute sinusitis?**

P In children with acute sinusitis

I1 short-course antibiotic therapy (5-7 days)

I2 prolonged antibiotic therapy (14 days)

C compared to antibiotic therapy for 10 days

O1 is equally effective in reducing the severity and/or duration of symptoms?

O2 does it modify the risk of recurrence?

O3 does it modify the risk of suppurative complications (orbital cellulitis, brain abscess, others)?

O4 does it modify the risk of non-suppurative complications (cavernous sinus thrombosis, others)?

O5 does it modify the risk of side effects?

1. **Which is the recommended second-line systemic antibiotic therapy for children with acute sinusitis?**

P In children with acute sinusitis and therapeutic failure of first-line therapy

I1 antibiotic therapy with cephalosporins

I2 antibiotic therapy with macrolides

I3 antibiotic therapy with fluoroquinolones

C compared to antibiotic therapy with amoxicillin-clavulanate

O1 is equally effective in reducing the severity and/or duration of symptoms?

O2 does it modify the risk of recurrence?

O3 does it modify the risk of suppurative complications (orbital cellulitis, brain abscess, others)?

O4 does it modify the risk of non-suppurative complications (cavernous sinus thrombosis, others)?

O5 does it modify the risk of side effects?

1. **Is systemic antibiotic treatment indicated in children with chronic sinusitis?**

P In children with chronic sinusitis

I systemic antibiotic therapy

C compared to anti-inflammatory therapy alone

O1 is more effective in reducing the severity and/or duration of symptoms?

O2 does it modify the risk of recurrence?

O3 does it modify the risk of suppurative complications (orbital cellulitis, brain abscess, others)?

O4 does it modify the risk of non-suppurative complications (cavernous sinus thrombosis, others)?

O5 does it modify the risk of side effects?

1. **Which is the first-line systemic antibiotic therapy indicated in children with chronic sinusitis?**

P In children with chronic sinusitis

I1 therapy with amoxicillin

I2 therapy with macrolides

I3 therapy with cephalosporins

I4 therapy with fluoroquinolones

C compared to antibiotic therapy with amoxicillin-clavulanate

O1 is equally effective in reducing the severity and/or duration of symptoms?

O2 does it modify the risk of recurrence?

O3 does it modify the risk of suppurative complications (orbital cellulitis, brain abscess, others)?

O4 does it modify the risk of non-suppurative complications (cavernous sinus thrombosis, others)?

O5 does it modify the risk of side effects?

1. **What is the duration of systemic antibiotic therapy in children with chronic sinusitis?**

P In children with chronic sinusitis

I1 10-day antibiotic therapy

I2 prolonged antibiotic therapy (>14 days)

C compared to antibiotic therapy for 14 days

O1 is equally effective in reducing the severity and/or duration of symptoms?

O2 does it modify the risk of recurrence?

O3 does it modify the risk of suppurative complications (orbital cellulitis, brain abscess, others)?

O4 does it modify the risk of non-suppurative complications (cavernous sinus thrombosis, others)?

O5 does it modify the risk of side effects?

1. **Which is the recommended treatment for a patient with uncomplicated sinusitis and penicillin allergy?**

P In children with uncomplicated sinusitis and penicillin allergy

I1 therapy with macrolides

I2 therapy with fluoroquinolones

I3 therapy with cephalosporins

C compared to therapy with amoxicillin

O1 is equally effective in reducing the severity and/or duration of symptoms?

O2 does it modify the risk of recurrence?

O3 does it modify the risk of suppurative complications (orbital cellulitis, brain abscess, others)?

O4 does it modify the risk of non-suppurative complications (cavernous sinus thrombosis, others)?

O5 does it modify the risk of side effects?

1. **Which antibiotic therapy is recommended for a child with a recurrence of uncomplicated acute sinusitis?**

P In children with recurrent uncomplicated acute sinusitis

I1 antibiotic therapy with cephalosporins

I2 antibiotic therapy with fluoroquinolones

I3 prolonged therapy with amoxicillin-clavulanate (for 14 days)

C compared to a new course of first-line antibiotics (amoxicillin-clavulanate for 10 days)

O1 is equally effective in reducing the severity and/or duration of symptoms?

O2 does it modify the risk of recurrence?

O3 does it modify the risk of suppurative complications (orbital cellulitis, brain abscess, others)?

O4 does it modify the risk of non-suppurative complications (cavernous sinus thrombosis, others)?

O5 does it modify the risk of side effects?

1. **Is antibiotic prophylaxis recommended in children with recurrent sinusitis?**

P In children with recurrent sinusitis

I antibiotic prophylaxis

C compared to no antibiotic therapy

O1 is it more effective in reducing the severity and/or duration of symptoms during recurrences?

O2 does it modify the risk of recurrence?

O3 does it modify the risk of suppurative complications (orbital cellulitis, brain abscess, others)?

O4 does it modify the risk of non-suppurative complications (cavernous sinus thrombosis, others)?

O5 does it modify the risk of side effects?

**Search strategy**

**PUBMED research 🡪 237**

(("paranasal sinuses"[MeSH Terms] OR ("paranasal"[All Fields] AND "sinuses"[All Fields]) OR "paranasal sinuses"[All Fields] OR "sinuses"[All Fields] OR "sinusal"[All Fields] OR "sinuse"[All Fields] OR "sinusitis"[MeSH Terms] OR "sinusitis"[All Fields] OR "sinusitides"[All Fields] OR ("rhinosinusal"[All Fields] OR "rhinosinusitis"[All Fields]) OR ("sinus"[Title/Abstract] AND "inflamm*"[Title/Abstract])) AND ("anti bacterial agents"[Pharmacological Action] OR "anti bacterial agents"[MeSH Terms] OR ("anti bacterial"[All Fields] AND "agents"[All Fields]) OR "anti bacterial agents"[All Fields] OR "antibiotic"[All Fields] OR "antibiotics"[All Fields] OR "antibiotic s"[All Fields] OR "antibiotical"[All Fields] OR ("anti bacterial agents"[Pharmacological Action] OR "anti bacterial agents"[MeSH Terms] OR ("anti bacterial"[All Fields] AND "agents"[All Fields]) OR "anti bacterial agents"[All Fields] OR "antibacterial"[All Fields] OR "antibacterials"[All Fields] OR "antibacterially"[All Fields]) OR ("aminoglycosid"[All Fields] OR "aminoglycosides"[MeSH Terms] OR "aminoglycosides"[All Fields] OR "aminoglycoside"[All Fields] OR "aminoglycosidic"[All Fields] OR "aminoglycosids"[All Fields]) OR ("fluoroquinolon"[All Fields] OR "fluoroquinolones"[MeSH Terms] OR "fluoroquinolones"[All Fields] OR "fluoroquinolone"[All Fields] OR "fluoroquinolonic"[All Fields] OR "fluoroquinolons"[All Fields]) OR ("cephalosporine"[All Fields] OR "cephalosporines"[All Fields] OR "cephalosporins"[MeSH Terms] OR "cephalosporins"[All Fields] OR "cephalosporin"[All Fields]) OR ("macrolid"[All Fields] OR "macrolides"[MeSH Terms] OR "macrolides"[All Fields] OR "macrolide"[All Fields] OR "macrolids"[All Fields]) OR ("amoxicillin"[MeSH Terms] OR "amoxicillin"[All Fields] OR "amoxicilline"[All Fields] OR "amoxicillins"[All Fields])) AND (("infant"[MeSH Terms] OR "child"[MeSH Terms] OR "adolescent"[MeSH Terms]) AND 2012/01/01:2022/12/31[Date - Publication])) OR (("paranasal sinuses"[MeSH Terms] OR ("paranasal"[All Fields] AND "sinuses"[All Fields]) OR "paranasal sinuses"[All Fields] OR "sinuses"[All Fields] OR "sinusal"[All Fields] OR "sinuse"[All Fields] OR "sinusitis"[MeSH Terms] OR "sinusitis"[All Fields] OR "sinusitides"[All Fields] OR ("rhinosinusal"[All Fields] OR "rhinosinusitis"[All Fields]) OR ("sinus"[Title/Abstract] AND "inflamm*"[Title/Abstract])) AND ("anti bacterial agents"[Pharmacological Action] OR "anti bacterial agents"[MeSH Terms] OR ("anti bacterial"[All Fields] AND "agents"[All Fields]) OR "anti bacterial agents"[All Fields] OR "antibiotic"[All Fields] OR "antibiotics"[All Fields] OR "antibiotic s"[All Fields] OR "antibiotical"[All Fields] OR ("anti bacterial agents"[Pharmacological Action] OR "anti bacterial agents"[MeSH Terms] OR ("anti bacterial"[All Fields] AND "agents"[All Fields]) OR "anti bacterial agents"[All Fields] OR "antibacterial"[All Fields] OR "antibacterials"[All Fields] OR "antibacterially"[All Fields]) OR ("aminoglycosid"[All Fields] OR "aminoglycosides"[MeSH Terms] OR "aminoglycosides"[All Fields] OR "aminoglycoside"[All Fields] OR "aminoglycosidic"[All Fields] OR "aminoglycosids"[All Fields]) OR ("fluoroquinolon"[All Fields] OR "fluoroquinolones"[MeSH Terms] OR "fluoroquinolones"[All Fields] OR "fluoroquinolone"[All Fields] OR "fluoroquinolonic"[All Fields] OR "fluoroquinolons"[All Fields]) OR ("cephalosporine"[All Fields] OR "cephalosporines"[All Fields] OR "cephalosporins"[MeSH Terms] OR "cephalosporins"[All Fields] OR "cephalosporin"[All Fields]) OR ("macrolid"[All Fields] OR "macrolides"[MeSH Terms] OR "macrolides"[All Fields] OR "macrolide"[All Fields] OR "macrolids"[All Fields]) OR ("amoxicillin"[MeSH Terms] OR "amoxicillin"[All Fields] OR "amoxicilline"[All Fields] OR "amoxicillins"[All Fields])) AND ("child"[MeSH Terms] OR "child"[All Fields] OR "children"[All Fields] OR "child s"[All Fields] OR "children s"[All Fields] OR "childrens"[All Fields] OR "childs"[All Fields] OR ("paediatrics"[All Fields] OR "pediatrics"[MeSH Terms] OR "pediatrics"[All Fields] OR "paediatric"[All Fields] OR "pediatric"[All Fields])) AND 2012/01/01:2022/12/31[Date - Publication])

Filters: Clinical Trial, Controlled Clinical Trial, Guideline, Meta-Analysis, Practice Guideline, Pragmatic Clinical Trial, Randomized Controlled Trial, Review, Systematic Review, Observational Study

**EMBASE research🡪 937**

('sinusitis'/exp OR sinusitis OR 'rhinosinusitis'/exp OR rhinosinusitis OR (sinus:ti,ab AND inflamm*:ti,ab)) AND ('antibiotics'/exp OR antibiotics OR 'antibacterial'/exp OR antibacterial OR 'aminoglycosides'/exp OR aminoglycosides OR 'fluoroquinolones'/exp OR fluoroquinolones OR 'cephalosporins'/exp OR cephalosporins OR 'macrolides'/exp OR macrolides OR 'amoxicillin'/exp OR amoxicillin) AND ([child]/lim OR [adolescent]/lim) AND [2012-2022]/py

OR

('sinusitis'/exp OR sinusitis OR 'rhinosinusitis'/exp OR rhinosinusitis OR (sinus:ti,ab AND inflamm*:ti,ab)) AND ('antibiotics'/exp OR antibiotics OR 'antibacterial'/exp OR antibacterial OR 'aminoglycosides'/exp OR aminoglycosides OR 'fluoroquinolones'/exp OR fluoroquinolones OR 'cephalosporins'/exp OR cephalosporins OR 'macrolides'/exp OR macrolides OR 'amoxicillin'/exp OR amoxicillin) AND ('child'/exp OR child OR 'pediatric'/exp OR pediatric) AND [2012-2022]/py

AND

([cochrane review]/lim OR [systematic review]/lim OR [meta analysis]/lim OR [controlled clinical trial]/lim OR [randomized controlled trial]/lim OR 'practice guideline' OR review OR 'clinical trial')

AND

Observational study

AND

AND ('Article'/it OR 'Article in Press'/it OR 'Conference Review'/it OR 'Review'/it)

**COCHRANE research 🡪 298**

#1 ((sinusitis* OR rhinosinusitis* OR (sinus AND inflamm*))):ti,ab,kw

#2 MeSH descriptor: [Sinusitis] explode all trees

#3 #1 OR #2

#4 (antibiotics OR antibacterial OR Aminoglycosides OR Fluoroquinolones OR Cephalosporins OR Macrolides OR Amoxicillin)

#5 MeSH descriptor: [Anti-Bacterial Agents] explode all trees

#6 MeSH descriptor: [Aminoglycosides] explode all trees

#7 MeSH descriptor: [Fluoroquinolones] explode all trees

#8 MeSH descriptor: [Cephalosporins] explode all trees

#9 MeSH descriptor: [Amoxicillin] explode all trees

#10 MeSH descriptor: [Anti-Bacterial Agents] explode all trees

#11 #4 OR #5 OR #6 OR #7 OR #8 OR #9 OR #10

#12 #3 AND #11

#13 ((child* OR pediatric*)):ti,ab,kw

#14 MeSH descriptor: [Child] explode all trees

#15 MeSH descriptor: [Adolescent] explode all trees

#16 #14 OR #15 OR #13

#17 #12 AND #16

Total 1472

Duplicates 208

**= 1264 screened**

*Excluded according to title and abstract* 🡪 1212

*Excluded according to full text* 🡪 42

*Included* 🡪 10

**Figure A2.1 Search algorithm records**

Records after removal of duplicates
(n = 1264)

## Screening

## Elegible

## Included

Records screened
(n = 1264)

## Identification

**Records identified through database search**

PUBMED n = 237

EMBASE n = 937
COCHRANE n = 298

Records excluded

(n=1212)

Full-text analysed

(n = 52)

Included studies
(n = 10)

Full-text excluded

(n = 42)

**Additional records identified from other sources**

(n = 0)

**Table A2.2 List of excluded articles with full text and reasons**

|  | DeMuri G, Wald ER. Acute bacterial sinusitis in children. Pediatr Rev. 2013;34(10):429-437; quiz. 437. | Article type (Narrative review) |
| --- | --- | --- |
|  | Forde R, Williams EW, Brown P, Mullings W. Acute complicated sinusitis: Ten years experience from the University Hospital of the West Indies (UHWI). West Indian Med J. 2017;66(2). | Article type (Narrative review) |
|  | Clement WA, Sooby P, Doherty C, Qayyum N, Irwin G. Acute isolated sphenoid sinusitis in children: A case series and systematic review of the literature. Int J Pediatr Otorhinolaryngol. 2021;140 | Non-relevant topic |
|  | Bártová I. Acute rhinosinusitis in children. Akutní rinosinusitidy u detí. 2012;13(6):372-378. | Article type (Narrative review) |
|  | Nocon CC, Baroody FM. Acute rhinosinusitis in children. Curr Allergy Asthma Rep. 2014;14(6):443. | Article type (Narrative review) |
|  | Brook I. Acute Sinusitis in Children. Pediatr Clin North Am. 2013;60(2):409-424. | Article type (Narrative review) |
|  | Abzug MJ. Acute sinusitis in children: do antibiotics have any role? J Infect. 2014;68:S33-7. | Article type (Narrative review) |
|  | Sabino HA, Valera FC, Aragon DC, et al. Amoxicillin-clavulanate for patients with acute exacerbation of chronic rhinosinusitis: a prospective, double-blinded, placebo-controlled trial. Int Forum Allergy Rhinol. 2017;7(2):135-142. | Adult population |
|  | Bellussi LM, Passali FM, Ralli M, De Vincentiis M, Greco A, Passali D. An overview on upper respiratory tract infections and bacteriotherapy as innovative therapeutic strategy. Eur Rev Med Pharmacol Sci. 2019;23(1):27-38. | Article type (Narrative review) |
|  | Brook I. Anaerobic bacteria in upper respiratory tract and head and neck infections: microbiology and treatment. Anaerobe. 2012;18(2):214-220. | Article type (Narrative review) |
|  | Grimprel E, Hentgen V, Lorrot M, Haas H, Cohen R. Antibiotherapy of severe ENT infections in children: Propositions of the French Group of Pediatric Infectious Diseases (PID) of the French Society of Pediatrics. Antibiothérapie des infections ORL sévères du nourrisson et de l’enfant: Propositions thérapeutiques du Groupe de pathologie infectieuse pédiatrique (GPIP) de la Société française de pédiatrie. 2013;20:e14-e19. | Non-English language |
|  | Koyama T, Hagiya H, Teratani Y, et al. Antibiotic prescriptions for Japanese outpatients with acute respiratory tract infections (2013–2015): A retrospective Observational Study. J Infect Chemother. 2020;26(7):660-666. | Non-relevant topic |
|  | Alves Galvão M, Rocha Crispino Santos M, Alves da Cunha A. Antibiotics for preventing suppurative complications from undifferentiated acute respiratory infections in children under five years of age. Cochrane Database of Systematic Reviews. 2016;(2). | Non-relevant topic |
|  | Kronman MP, Zhou C, Mangione-Smith R. Bacterial prevalence and antimicrobial prescribing trends for acute respiratory tract infections. Pediatrics. 2014;134(4):e956-e965. | Non-relevant topic |
|  | Chandran SK, Higgins TS. Chapter 5: Pediatric rhinosinusitis: definitions, diagnosis and management--an overview. Am J Rhinol Allergy. 2013;27:S16-19. | Article type (Narrative review) |
|  | Quintanilla-Dieck L, Lam DJ. Chronic Rhinosinusitis in Children. Curr Treat Options Pediatr. 2018;4(4):413-424. | Article type (Narrative review) |
|  | Rose AS, Thorp BD, Zanation AM, Ebert CS. Chronic Rhinosinusitis in Children. Pediatr Clin North Am. 2013;60(4):979-991. | Article type (Narrative review) |
|  | Chandy Z, Ference E, Lee JT. Clinical Guidelines on Chronic Rhinosinusitis in Children. Curr Allergy Asthma Rep. 2019;19(2). | Article type (Narrative review) |
|  | DeMuri GP, Wald ER. Clinical practice. Acute bacterial sinusitis in children. N Engl J Med. 2012;367(12):1128-1134. | Article type (Narrative review) |
|  | Lopatin AS, Ivanchenko OA, Soshnikov SS, Mullol J. Cyclamen europaeum improves the effect of oral antibiotics on exacerbations and recurrences of chronic rhinosinusitis: a real-life observational study (CHRONOS). Acta Otorhinolaryngol Ital. 2018;38(2):115-123. | Non-relevant topic |
|  | Peters AT, Spector S, Hsu J, et al. Diagnosis and management of rhinosinusitis: A practice parameter update. Ann Allergy Asthma Immunol. 2014;113(4):347-385. | Article type (guideline) |
|  | Improving Antibiotic Prescribing for Pediatric Respiratory Infections by Family Physicians With Peer Comparison. https://clinicaltrials.gov/show/NCT04588376. Published online 2020. | Non-relevant topic |
|  | Dekker AR, Verheij TJ, van der Velden AW. Inappropriate antibiotic prescription for respiratory tract indications: most prominent in adult patients. Fam Pract. 2015;32(4):401-407. | Non-relevant topic |
|  | Speakman J, Srinivasand S, Taggart L, et al. Index of suspicion. Pediatr Rev. 2013;34(10):465-473. | Non-relevant topic |
|  | Esposito S, Rosazza C, Sciarrabba CS, Principi N. Inhaled Antibiotic Therapy for the Treatment of Upper Respiratory Tract Infections. J Aerosol Med Pulm Drug Deliv. 2017;30(1):14-19. | Article type (Narrative review) |
|  | Mori F, Fiocchi A, Barni S, et al. Management of acute rhinosinusitis. Pediatr Allergy Immunol. 2012;23:27-31. | Article type (Narrative review) |
|  | Cazzavillan A, Castelnuovo P, Berlucchi M, et al. Management of chronic rhinosinusitis. Pediatr Allergy Immunol. 2012;23:32-44. | Article type (Narrative review) |
|  | Abudinen-Vasquez S, Marin MN. Management of pediatric head and neck infections in the emergency department. Pediatr Emerg Med Pract. 2020;17(11):1-24. | Article type (Narrative review) |
|  | Shimizu T, Suzaki H. Past, present and future of macrolide therapy for chronic rhinosinusitis in Japan. Auris Nasus Larynx. 2016;43(2):131-136. | Article type (Narrative review) |
|  | Magit A. Pediatric rhinosinusitis. Otolaryngol Clin North Am. 2014;47(5):733-746. | Article type (Narrative review) |
|  | Pavez D, Pérez R, Cofré J, Rodríguez J. Recommendations for diagnosis and antimicrobial treatment of acute bacterial rhinosinusitis in pediatrics. Recomendaciones para el diagnóstico y tratamiento antimicrobiano de la rinosinusitis aguda bacteriana en pediatría. 2019;36(1):78-82. | Non-English language |
|  | DeCastro A, Mims L, Hueston WJ. Rhinosinusitis. Prim Care. 2014;41(1):47-61. | Adult population |
|  | Zhao SR, Griffin MR, Patterson BL, et al. Risk Factors for Outpatient Use of Antibiotics in Children with Acute Respiratory Illnesses. South Med J. 2017;110(3):172-180. | Non-relevant topic |
|  | Mandal R, Patel N, Ferguson BJ. Role of antibiotics in sinusitis. Curr Opin Infect Dis. 2012;25(2):183-192. | Article type (Narrative review) |
|  | Dawson-Hahn EE, Mickan S, Onakpoya I, et al. Short-course versus long-course oral antibiotic treatment for infections treated in outpatient settings: a review of systematic reviews. Fam Pract. 2017;34(5):511-519. | Adult population |
|  | Brook I. The role of antibiotics in pediatric chronic rhinosinusitis. Laryngoscope Investig Otolaryngol. 2017;2(3):104-108. | Article type (Narrative review) |
|  | Mösges R, Desrosiers M, Arvis P, Heldner S. Characterisation of patients receiving moxifloxacin for acute bacterial rhinosinusitis in clinical practice: results from an international, observational cohort study. PLoS One. 2013;8(4):e61927. | Study type (post-marketing study) |
|  | R Deepa, R Jyothi, H P Pundarikaksha, B Jagannath. A study on the drug prescribing pattern in para-nasal sinusitis at a tertiary care hospital. [Natl J Physiol Pharm Pharmacol](https://www.bibliomed.org/?jtt=2320-4672). 2014; 4(3): 182-186 | Non-relevant topic |
|  | Seresirikachorn K, Chetthanon T, Suwansirisuk T, et al. Low-dose macrolides for treating pediatric rhinosinusitis: A retrospective study and literature review. SAGE Open Med. 2020;8: Published 2020 Jun 30. | Non-relevant topic |
|  | Zhao Q, Yu L, Jin P, Ma W, Duan S, Luo H. A comprehensive investigation of the demographics, treatments, comorbidities, and disease burden of chronic rhinosinusitis with nasal polyposis patients: a descriptive analysis. Ann Transl Med. 2022 Feb;10(3):150. | Non-relevant topic |
|  | Veskitkul J, Vichyanond P, Pacharn P, Visitsunthorn N, Jirapongsananuruk O. Clinical characteristics of recurrent acute rhinosinusitis in children. Asian Pac J Allergy Immunol. 2015 Dec;33(4):276-80. | Study type (no available control cohort) |
|  | Poachanukoon O, Tangsathapornpong A, Tanuchit S. A Comparison of Cefditoren Pivoxil 8-12 mg/kg/day and Cefditoren Pivoxil 16-20 mg/kg/day in Treatment of Children With Acute Presumed Bacterial Rhinosinusitis: A Prospective, Randomized, Investigator-Blinded, Parallel-Group Study. Clin Exp Otorhinolaryngol. 2015 Jun;8(2):129-35. | Non-relevant topic |
