# Supplementary material for: Treatment of sinusitis in children: an Italian intersociety consensus (SIPPS-SIP-SITIP-FIMP-SIAIP-SIMRI-SIM-FIMMG)
Source: Ital J Pediatr. 2025 Mar 26;51:102. doi: 10.1186/s13052-025-01868-1 (PMC11948864; doi:10.1186/s13052-025-01868-1)
Supplement: Supplementary file 2 — Supplementary Material 2 [file 13052_2025_1868_MOESM2_ESM.docx]

**PICO 1.**

**Question: Antibiotic versus standard of care or placebo for acute sinusitis therapy**

**Setting:** Outpatients

**Bibliography:**

| **Certainty assessment** | | | | | | | **№ of patients** | | **Effect** | | **Confidence** | **Importance** |
| --- | --- | --- | --- | --- | --- | --- | --- | --- | --- | --- | --- | --- |
| **№ of studies** | **Study design** | **Risk of distortion** | **Lack of reproducible results** | **Lack of generalizability** | **Imprecision** | **Further considerations** | **antibiotics** | **standard of care or placebo** | **Relative (95% CI)** | **Absolute (95% CI)** |  |  |
| **Improvement in symptoms at 10-14 days from the start of therapy (clinical score) (median follow-up: 14 days)** | | | | | | | | | | | | |
| 4 | randomized studies | not important^a^ | not important | serious^a^ | not important | none | 227/425 (53.4%) | 155/425 (36.5%) | **RR 1.24** (1.08 a 1.42) | 87 more per 1,000  (from 28 more to 154 more) | ⨁⨁⨁◯ Moderate | IMPORTANT |
| 1 | randomized studies | not important | not important | not important | not important | none | 40/80 (50.0%) | 40/80 (50.0%) | **RR 1.01** (0.74 a 1.39) | **7 more per 1.000** (from 132 less to 197 more) | ⨁⨁⨁⨁ High | IMPORTANT |
| 1 | randomized studies | serious^c^ | not important | serious | serious^d^ | Strongly suspected publication bias  All plausible residual confounders could reduce the demonstrated effect | 11/57 (19.3%) | 46/57 (80.7%) | not estimable |  | ⨁◯◯◯ Very low | NOT IMPORTANT |
| 4 | randomized studies | not important | serious | serious | not important | none | 227/386 (58.8%) | 159/386 (41.2%) | **RR 1.34** (1.13 a 1.59) | **141 more per 1.000** (from 54 more to 244 more) | ⨁⨁◯◯ Low | IMPORTANT |
| **Clinical improvement at 21 days from the start of therapy (clinical score, radiography, and respiratory function tests) (follow-up: 21 days)** | | | | | | | | | | | | |
| 1 | randomized studies | serious^b^ | not important | not important | not important | none | 45/91 (49.5%) | 46/91 (50.5%) | not estimable |  | ⨁⨁⨁◯ Moderate | IMPORTANT |
|  | | | | | | | | | | | | |
| **effectiveness of antibiotic therapy in preventing the development of complications** | | | | | | | | | | | | |
| 1 | Observational studies | not important | not important | not important | not important | none | 8/23493 (0.0%) | 5/2797 (0.2%) | **OR 0.19** (0.06 a 0.58) | **1 less per 1.000** (from 2 less to 1 less) | ⨁⨁◯◯ Low | NOT IMPORTANT |
| **Antibiotic side effects vs. placebo (median follow-up: 14 days)** | | | | | | | | | | | | |
| 5 | randomized studies | Serious ^e^ | Serious ^f^ | Serious ^g^ | not important | none | 59/255 (23.1%) | 23/184 (12.5%) | **RR 1.85** (1.19 a 2.88) | **106 more per 1.000** (from 24 more 235 more) | ⨁◯◯◯ Very low | NOT IMPORTANT |

**CI:** Confidence interval; **OR:** Odds ratio; **RR:** Risk ratio

#### Explanations

a. Risks for internal bias were thought to be small for each study, but external bias is potentially significant.

b. Randomization not performed by computer

c. Funded by a pharmaceutical company

d. The method of administering therapy vs. placebo in the groups is unclear

e. Not all studies are double-blind

f. High heterogeneity

g. Use of different antibiotics (compared to placebo)

**Quesiton: Antibiotic vs. placebo or anti-inflammatory therapy for acute sinusitis with symptoms lasting 30-90 days (subacute)**

**Setting:** Outpatients

**Bibliography:**

| **Certainty assessment** | | | | | | | **№ of patients** | | **Effect** | | **Confidence** | **Importance** |
| --- | --- | --- | --- | --- | --- | --- | --- | --- | --- | --- | --- | --- |
| **№ of studies** | **Study design** | **Risk of distortion** | **Lack of reproducible results** | **Lack of generalizability** | **Imprecision** | **Further considerations** | **antibiotics** | **standard of care or placebo** | **Relative (95% CI)** | **Absolute (95% CI)** |  |  |
| **Radiographic and/or symptomatic response (follow-up: interval of 3 to 6 weeks; assessed with: X-ray).** | | | | | | | | | | | | |
| 1 | randomized studies | not important | not important | serious | not important | none | 55/77 (71.4%) | 12/19 (63.2%) | **RR 1.1310** (0.7801 a 1.6395) | **83 more per 1.000** (from 139 less to 404 more) | ⨁⨁⨁◯ Moderate | IMPORTANT |

**CI:** Confidence interval; **RR:** Risk ratio

**PICO 3.**

**Question: Antibiotic 1 versus antibiotic 2 for the treatment of acute sinusitis?**

**Setting:** Outpatients

**Bibliography:**

| **Certainty assessment** | | | | | | | **№ of patients** | | **Effect** | | **Confidence** | **Importance** |
| --- | --- | --- | --- | --- | --- | --- | --- | --- | --- | --- | --- | --- |
| **№ of studies** | **Study design** | **Risk of distortion** | **Lack of reproducible results** | **Lack of generalizability** | **Imprecision** | **Further considerations** | **antibiotics** | **standard of care or placebo** | **Relative (95% CI)** | **Absolute (95% CI)** |  |  |
| **Clinical improvement: amoxicillin vs. cephalosporin (follow-up: interval 10 to 14 days)** | | | | | | | | | | | | |
| 2 | randomized studies | serious^a^ | serious^a^ | serious^a^ | serious^a^ | none | 84/99 (84.8%) | 72/89 (80.9%) | **RR 1.05** (0.92 a 1.19) | **39 more per 1.000** (from 65 less ro 158 more) | ⨁◯◯◯ Very low | NOT IMPORTANT |
| **Clinical improvement: macrolide vs. cephalosporin (follow-up: 28 days)** | | | | | | | | | | | | |
| 1 | randomized studies | serious^b^ | Not important | serious^c^ | serious^c^ | none | 48/50 (96.0%) | 142/150 (94.7%) | **RR 1.01** (0.95 a 1.08) | **13 more per 1.000** (from 50 less to 81 more) | ⨁◯◯◯ Very low | NOT IMPORTANT |
| **Clinical improvement: amoxicillin-clavulanate vs. cefuroxime (follow-up: 14 days)** | | | | | | | | | | | | |
| 1 | randomized studies | serious^b^ | serious^d^ | serious^d,e^ | serious^c,d^ | none | 30/42 (71.4%) | 49/57 (86.0%) | **RR 0.83** (0.67 a 1.03) | **145 less per 1.000** (from 285 less to 29 more) | ⨁◯◯◯ Very low | NOT IMPORTANT |

**CI:** Confidence interval; **RR:** Risk ratio

#### Explanations

a. Absence of a placebo group; different and non-standardized treatments

b. Not double-blinded

c. Limited population

d. Aggregate data from adults and children

e. children > 12

**Question: Systemic antibiotic therapy compared to amoxicillin-clavulanate for subacute sinusitis (30-90 days)**

**Setting:** Outpatients

**Bibliography:**

| **Certainty assessment** | | | | | | | **№ of patients** | | **Effect** | | **Confidence** | **Importance** |
| --- | --- | --- | --- | --- | --- | --- | --- | --- | --- | --- | --- | --- |
| **№ of studies** | **Study design** | **Risk of distortion** | **Lack of reproducible results** | **Lack of generalizability** | **Imprecision** | **Further considerations** | **antibiotics** | **standard of care or placebo** | **Relative (95% CI)** | **Absolute (95% CI)** |  |  |
| **Clinical improvement (median 3-week follow-up; assessed using numeric scales reported by parents)** | | | | | | | | | | | | |
| 3 | randomized studies | Very serious ^a,b,c^ | Very serious ^c,d^ | serious^a,b^ | extremely serious^a,b,d^ | All plausible residual confounders could reduce the demonstrated effect | 73/98 (74.5%) | 51/72 (70.8%) | **RR 0.9509** (0.7878 a 1.1477) | **35 less per 1.000** (from 150 less to 105 more) | ⨁◯◯◯ Very low | IMPORTANT |

**CI:** Confidence interval; **RR:** Risk ratio

#### Explanations

a. Heterogeneity of the target population (<2 aa vs 2-12 vs 5-16)

b. Heterogeneity of the antibiotic molecules used compared to Amoxicillin-Clavulanate

c. Use of non-standardized clinical assessment scales

d. Use of poorly standardized complementary therapies (corticosteroids, saline solution)

**PICO 7-8.**

**Question: Is systemic antibiotic treatment indicated in children with chronic sinusitis?**

**Setting:** Outpatients

**Bibliography:**

| **Certainty assessment** | | | | | | | **№ of patients** | | **Effect** | | **Confidence** | **Importance** |
| --- | --- | --- | --- | --- | --- | --- | --- | --- | --- | --- | --- | --- |
| **№ of studies** | **Study design** | **Risk of distortion** | **Lack of reproducible results** | **Lack of generalizability** | **Imprecision** | **Further considerations** | **antibiotics** | **standard of care or placebo** | **Relative (95% CI)** | **Absolute (95% CI)** |  |  |
| **Nasal symptoms (median 14-week follow-up; assessed using a visual analog scale)** | | | | | | | | | | | | |
| 1 | observational studies | serious^a^ | serious^a^ | very serious^a^ | extremely serious^a^ | All potential residual confounders could diminish the demonstrated effect | 6/6 (100.0%) | - | - | - | ⨁◯◯◯ Very low | NOT IMPORTANT |
| **Nasal symptoms (median 14-day follow-up; assessed with clinical evaluation + radiology)** | | | | | | | | | | | | |
| 1 | randomized studies | serious^a,b^ | not important | not important | Serious ^b^ | none | 24/37 (64.9%) | 20/38 (52.6%) | **RR 1.232** (0.839 a 1.809) | **122 more per 1.000** (from 85 less to 426 more) | ⨁⨁◯◯ Low | NOT IMPORTANT |

**CI:** Confidence interval; **RR:** Risk ratio

#### Explanations

a. Drug dose not specified in the text, follow-up period not defined

b. Outcome at 12 weeks not reported (as stated in the methods)

**PICO 12.**

**Question:** Is antibiotic prophylaxis recommended in children with recurrent sinusitis?

**Setting:** Outpatients

**Bibliography:**

| **Certainty assessment** | | | | | | | **№ of patients** | | **Effect** | | | **Confidence** | **Importance** |
| --- | --- | --- | --- | --- | --- | --- | --- | --- | --- | --- | --- | --- | --- |
| **№ of studies** | **Study design** | **Risk of distortion** | **Lack of reproducible results** | **Lack of generalizability** | **Imprecision** | **Further considerations** | **antibiotics** | **standard of care or placebo** | **Relative (95% CI)** | **Absolute (95% CI)** | |  |  |
| **Number of episodes of recurrent acute rhinosinusitis (RARS) in 12 months** | | | | | | | | | | | | | |
| 1 | randomized studies | not important | Not applicable | serious^a^ | serious^b^ | none | -3.2 ±2.6 | 0.6 ± 2.0 | -3.8 ± 0.7 | -5.3 to -2.3 | 3 | |  |

#### Explanations:

####

a. The severity assessment was deduced from a patient-reported symptom score (not always objectively verified by the physician), and the duration of symptoms was not evaluated

b. Limited number of patients and events
