# Supplementary material for: Treatment of sinusitis in children: an Italian intersociety consensus (SIPPS-SIP-SITIP-FIMP-SIAIP-SIMRI-SIM-FIMMG)
Source: Ital J Pediatr. 2025 Mar 26;51:102. doi: 10.1186/s13052-025-01868-1 (PMC11948864; doi:10.1186/s13052-025-01868-1)
Supplement: Supplementary file 3 — Supplementary Material 3 [file 13052_2025_1868_MOESM3_ESM.docx]

| **Question 1: Is antibiotic treatment indicated for a child with uncomplicated acute sinusitis?** | P: In children with acute sinusitis without specific risk factors,  I1: Is topical antibiotic therapy  I2: Is systemic antibiotic therapy  C: Compared to anti-inflammatory therapy alone,  O1: Equally effective in reducing the severity and/or duration of symptoms?  O2: Does it modify the risk of recurrences?  O3: Does it modify the risk of suppurative complications (orbital cellulitis, brain abscess, other)?  O4: Does it modify the risk of non-suppurative complications (cavernous sinus thrombosis, other)?  O5: Does it modify the risk of side effects? |
| --- | --- |

| **Systematic review** | **Population and Purpose of the SR** | **Results** | **Conclusion** |
| --- | --- | --- | --- |
| **Michael J. Smith, 2013** | - 4 RCTs (392 children): Efficacy of antibiotic therapy in children with acute sinusitis (not all studies excluded those with specific risk factors), comparing antibiotic therapy with placebo. - 1 RCT (96 children aged 2 to 16 years): Efficacy of systemic antibiotic therapy in children with acute sinusitis with symptoms lasting 30-90 days and radiological abnormalities of the paranasal sinuses assessed by facial X-ray, comparing antibiotic therapy with placebo. (Wang Dolhman et al. 1993) | - 2 RCTs favorable to treatment (Wald ER et al. 2009 #6; Wald ER et al. 1986 #7), 2 RCTs (Garbutt JM et al. 2001 #8; Kristo A et al. 2005 #9) showing no significant difference between treatment and placebo. Qualitative analysis suggests that there may be some clinical characteristics (such as greater severity of the condition at presentation) that could help identify patients who might benefit from antibiotic therapy. - No difference in terms of efficacy | - Data on the proper management of sinusitis are currently limited and do not provide clear guidance on the correct use of antibiotic therapy. Additionally, there is a need for updated guidelines to identify children who require antibiotic therapy from the outset. - Regarding acute sinusitis with symptoms lasting 30-90 days, the available data are limited and of poor methodological quality |
| **Cronin et al.; 2013** | Pediatric patients (0-18 years) with acute bacterial sinusitis.  Evaluate, based on the available literature, the efficacy of antibiotic therapy compared to placebo in children with acute sinusitis (Garbutt JM et al., 2001 #27; Kristo A et al., 2005 #28; Wald ER et al., 1986 #29; Wald ER et al., 2009 #30). | The quantitative analysis of the data showed an improvement in symptoms 14 days after the start of therapy (10 days in one of the included studies) in patients treated with antibiotics compared to the placebo group (OR 2.0 (95% CI 1.16 to 3.47; I2 = 14.8%)). None of the patients treated with placebo developed significant complications secondary to sinusitis. The meta-analysis suggests the effectiveness of antibiotic therapy in the treatment of acute sinusitis. | Although the results of the meta-analysis support the use of antibiotic therapy, the authors, in accordance with English guidelines, advocate for a conservative approach, recommending against the routine prescription of antibiotics and limiting it to selected cases |
| **Axiotakis et al.; 2022** | Pediatric patients (0-18 years) with acute bacterial sinusitis. Characterize the incidence of adverse events related to the use of oral antibiotic therapy (Wald ER et al. 2009 #4; Wald ER et al. 1986 #13; Garbutt JM et al., 2001 #16; Kristo A et al., 2005 #18; Ragab et al., 2015 #21). | Diarrhea is the most common side effect in patients receiving antibiotic therapy as well as in those in the placebo group. No significant differences were reported between the two groups (OR 2.20 (CI: 0.86-5.61, I2 = 65)). The incidence of skin rashes is, however, low. | Currently, the literature on adverse events related to antibiotic use in children with acute sinusitis is limited. However, although potential drug reactions should not be overlooked, such events do not appear to be significantly higher compared to children not receiving antibiotic therapy. |

| **Study**  **(Author, Year)** | **Study Design** | **Population (Number, Country, Setting, Condition)** | **Intervention/Exposure** | **Primary Outcome** | **Effect Measures** | **Secondary Outcomes** | **Follow-up** | **Results** | **Funding** |
| --- | --- | --- | --- | --- | --- | --- | --- | --- | --- |
| **Tugrul et al.; 2014** | RCT double blind | Turkey. 91 children (5-18 years) with uncomplicated acute sinusitis without risk factors, evaluated on an outpatient basis. | Low-pressure nasal washes with saline solution and fluticasone propionate for three weeks | Evaluate the efficacy of nasal washes with saline solution and fluticasone propionate in the treatment of acute sinusitis in pediatric patients compared to the administration of antibiotics (amoxicillin-clavulanic acid) and nasal decongestants. | T-test  Anova test  Test chi-quadrato |  | Clinical follow-up at 7, 14, and 21 days from the start of therapy. At 21 days from the start of therapy: • Radiological assessment (maxillary sinus X-ray) • Peak nasal inspiratory flow (PNIF) • Blood tests (complete blood count, C-reactive protein, erythrocyte sedimentation rate) | In the group treated with nasal washes and fluticasone, a more rapid improvement in symptoms was recorded (p<0.05 at 7 days), with no significant differences at 21 days from the start of therapy. No significant differences were reported between the two groups in the radiological and blood tests at 21 days (p=0.072 and p>0.05). A more pronounced (but not significant) improvement in PNIF was observed in the group treated with nasal washes and fluticasone. | Not declared |
| **Khoshdel et al.; 2014** | RCT double blind | Iran. 80 children (4-15 years) with non-severe, uncomplicated acute sinusitis without risk factors, evaluated on an outpatient basis | Oral amoxicillin (80 mg/kg/day) in 3 doses for 14 days, with nasal washes using saline solution (for 5 days) and phenylephrine (for two days) | Evaluate the efficacy of antibiotic therapy with high doses of amoxicillin and nasal washes compared to nasal washes alone in the management of acute sinusitis in children. | Fisher exact Test  T-test |  | Clinical follow-up on days 3, 14, 21, and 28 after the start of antibiotic therapy. | Three days after the start of therapy, the group treated with amoxicillin showed a significant improvement in symptoms compared to the control group (p=0.001). However, no further significant differences between the two groups were reported during the remaining follow-up (p > 0.05). All patients were recovered by 21 and 28 days after the start of therapy. | Shahrekord  Medical University of Sciences |
| **Meltzer et al.; 2012** | RCT double blind | USA/Europe.  981 patients aged > 12 years (57 aged 12-18 years) with uncomplicated, non-severe acute sinusitis, evaluated on an outpatient basis. | Mometasone furoate nasal spray 200 µg once daily or 200 µg twice daily for 15 days | Evaluate the efficacy of mometasone furoate nasal spray in reducing symptoms associated with acute sinusitis compared to amoxicillin (500 mg three times daily) and compared to placebo. | Ancova test  T-test |  | Patient-completed questionnaire twice daily (morning and evening) for up to 14 days after the end of antibiotic therapy | Mometasone furoate nasal spray 200 µg twice daily significantly reduces symptoms compared to amoxicillin (p=0.0040) and placebo (p < 0.0001) in patients with acute sinusitis. | Schering-Plough, now  Merck Sharp & Dohme Corp., Whitehouse Station, NJ, USA. |
| **Cushen, 2020** | Cohort, retrospective | UK  Number = 0-20 years, 26,290 with uncomplicated acute sinusitis diagnosed on an outpatient basis. | Antibiotic therapy not specified | Evaluate the efficacy of antibiotic therapy in preventing the development of complications. | Clinica records screening | / | 1 months | No pediatric cases of brain abscesses following sinusitis.  Incidence of orbital cellulitis: 4.94 (95% CI = 2.89-8.46) per 10,000 episodes of sinusitis in the population; antibiotic therapy significantly reduces the risk of orbital cellulitis compared to the placebo group (OR 0.19, 95% CI 0.06-0.58). However, routine prescription is not recommended due to the rarity of the event (NNT 691, 95% CI = 260-2695). | no |

| **Question 2: What first-line topical antibiotic therapy is indicated for a child with uncomplicated acute sinusitis?** | P: In children with acute sinusitis without specific risk factors,  I1: Topical antibiotic therapy with aminoglycosides  I2: Topical antibiotic therapy with fluoroquinolones  C: Compared to topical antibiotic therapy with cephalosporins,  O1: Is it equally effective in reducing the severity and/or duration of symptoms?  O2: Does it modify the risk of recurrences?  O3: Does it modify the risk of suppurative complications (orbital cellulitis, brain abscess, other)?  O4: Does it modify the risk of non-suppurative complications (cavernous sinus thrombosis, other)?  O5: Does it modify the risk of side effects? |
| --- | --- |

Insufficient literature to answer the question.

| **Question 3: What first-line systemic antibiotic therapy is indicated for a child with uncomplicated acute sinusitis?** | P: In children with acute sinusitis without specific risk factors,  I1: Antibiotic therapy with amoxicillin  I2: Antibiotic therapy with macrolides  I3: Antibiotic therapy with cephalosporins  I4: Antibiotic therapy with fluoroquinolones  C: Compared to antibiotic therapy with amoxicillin-clavulanic acid,  O1: Is it equally effective in reducing the severity and/or duration of symptoms?  O2: Does it modify the risk of recurrences?  O3: Does it modify the risk of suppurative complications (orbital cellulitis, brain abscess, other)?  O4: Does it modify the risk of non-suppurative complications (cavernous sinus thrombosis, other)?  O5: Does it modify the risk of side effects? |
| --- | --- |

| **Systematic review** | **Population and Purpose of the SR** | **Results** | **Conclusion** |
| --- | --- | --- | --- |
| **Michael J. Smith, 2013** | - 5 RCTs (Poachanukoon O. et al, 2008 #12; Simon MW, 1999 #13; Ficnar B. et al, 1997 #14; Careddu P. et al, 1993 #15; Wald ER et al., 1984 #16) comparing different antibiotic therapies in children with acute sinusitis (placebo group not included). - 3 RCTs (Wang Dolhman et al. 1993; El-Hennawi et al. 2006; Ng et al. 2000) comparing different antibiotic therapies in the systemic treatment of children with acute sinusitis with symptoms lasting 30-90 days. | - No RCT demonstrates the advantage of one antibiotic therapy over another, with an overall cure or improvement rate > 80% - No RCT demonstrates the advantage of one systemic antibiotic therapy over another in the treatment of acute sinusitis with symptoms lasting 30-90 days. One RCT (El-Hennawi et al. 2006) shows the benefit of targeted antibiotic therapy, based on nasal mucus culture, compared to empirical therapy with amoxicillin-clavulanic acid. | - Data on the proper management of sinusitis are currently limited and do not provide clear guidance on the correct use of antibiotic therapy. Additionally, there is a need for updated guidelines to identify children who require antibiotic therapy from the outset. |

| **Study**  **(Author, Year)** | **Study Design** | **Population (Number, Country, Setting, Condition)** | **Intervention/Exposure** | **Primary Outcome** | **Effect Measures** | **Secondary Outcomes** | **Follow-up** | **Results** | **Funding** |
| --- | --- | --- | --- | --- | --- | --- | --- | --- | --- |
| **Lari AR. et al.; 2012** | RCT | Iran.  99 patients aged > 12 years with uncomplicated acute sinusitis, evaluated on an outpatient basis. | Cefuroxime 250 mg twice daily for 10 days or amoxicillin-clavulanic acid 500/125 mg three times daily for 10 days | Compare the efficacy of amoxicillin-clavulanic acid and cefuroxime in the treatment of acute bacterial sinusitis. | T-test |  | Clinical follow-up during therapy: from 6 to 8 days after the start.  Clinical follow-up from 2 to 5 days after the end of antibiotic therapy. | No significant difference between amoxicillin-clavulanic acid and cefuroxime in the treatment of acute bacterial sinusitis (p > 0.05). | Not declare |

| **Question 4: What dosage of first-line systemic antibiotic therapy is indicated for a child with uncomplicated acute sinusitis?** | P: In children with acute sinusitis without specific risk factors,  I: Low-dose amoxicillin-clavulanic acid (50 mg/kg/day) in 2-3 daily doses  C: Compared to high-dose amoxicillin-clavulanic acid (90 mg/kg/day) in 2-3 daily doses  O1: Is it equally effective in reducing the severity and/or duration of symptoms?  O2: Does it modify the risk of recurrences?  O3: Does it modify the risk of suppurative complications (orbital cellulitis, brain abscess, other)?  O4: Does it modify the risk of non-suppurative complications (cavernous sinus thrombosis, other)?  O5: Does it modify the risk of side effects? |
| --- | --- |

Insufficient literature to answer the question.

| **Question 5: What is the duration of oral systemic antibiotic therapy with amoxicillin-clavulanic acid for a child with uncomplicated acute sinusitis?** | P: In children with acute sinusitis,  I1: Short-course antibiotic therapy (5-7 days)  I2: Prolonged antibiotic therapy (14 days)  C: Compared to 10-day antibiotic therapy  O1: Is it equally effective in reducing the severity and/or duration of symptoms?  O2: Does it modify the risk of recurrences?  O3: Does it modify the risk of suppurative complications (orbital cellulitis, brain abscess, other)?  O4: Does it modify the risk of non-suppurative complications (cavernous sinus thrombosis, other)?  O5: Does it modify the risk of side effects? |
| --- | --- |

Insufficient literature to answer the question.

| **Question 6: What second-line systemic antibiotic therapy is indicated for a child with acute sinusitis?** | P: In children with acute sinusitis who have failed first-line therapy,  I1: Antibiotic therapy with cephalosporins  I2: Antibiotic therapy with macrolides  I3: Antibiotic therapy with fluoroquinolones  C: Compared to antibiotic therapy with amoxicillin-clavulanic acid  O1: Is it equally effective in reducing the severity and/or duration of symptoms?  O2: Does it modify the risk of recurrences?  O3: Does it modify the risk of suppurative complications (orbital cellulitis, brain abscess, other)?  O4: Does it modify the risk of non-suppurative complications (cavernous sinus thrombosis, other)?  O5: Does it modify the risk of side effects? |
| --- | --- |

Insufficient literature to answer the question.

|  | |  |  |  |
| --- | --- | --- | --- | --- |
| **Question 7: Is systemic antibiotic treatment indicated for a child with chronic sinusitis?** | **P**Nei bambini con sinusite cronica **I**la terapia antibiotica sistemica **C**  rispetto alla sola terapia antinfiammatoria  **O1**è più efficace nel ridurre la gravità e/o la durata della sintomatologia? **O2**modifica il rischio di recidive? **O3** modifica il rischio di complicanze suppurative (cellulite orbitaria, ascesso cerebrale, altro)? **O4** modifica il rischio di complicanze non suppurative (trombosi seni cavernosi, altro)? **O5** modifica il rischio di effetti collaterali? | | | |

**.**

| **Systematic review** | **Population and Purpose of the SR** | **Results** | **Conclusion** |
| --- | --- | --- | --- |
| Head K. et al. Systemic and topical antibiotics for chronic rhinosinusitis. | **Population:** Adults aged 20 to 70 years, plus a single pediatric study with 79 children aged 2-12 years.  **Antibiotic Therapy:** 3 months of therapy with macrolides or tetracyclines.  **Comparisons:**   - Antibiotic vs. placebo - Antibiotic + intranasal saline vs. placebo + saline - Antibiotic vs. nasal steroid - Antibiotic vs. oral steroid   **Outcome:** Assessment using the SNOT-20 (Sinus Nasal Outcome Test-20). | **Antibiotic vs. placebo:** In 3 studies, there is evidence of a reduction in SNOT-20 scores in the treated group at the end of the treatment, but this evidence is not confirmed 3 months after the end of therapy.  **Antibiotic + intranasal saline vs. placebo + intranasal saline:** No difference between antibiotic and placebo.  **Antibiotic vs. nasal steroid:** Uncertain evidence.  **Antibiotic vs. oral steroid:** Uncertain evidence. | The evidence comes from 5 studies, including only one pediatric study (Otten et al. 1994 – 79 patients). The results are not stratified by age. The heterogeneity of the antibiotic treatments used is not commented on. |

| **Question 8: What first-line systemic antibiotic therapy is indicated for a child with chronic sinusitis?** | P: In children with chronic sinusitis,  I1: Therapy with amoxicillin  I2: Therapy with macrolides  I3: Therapy with cephalosporins  I4: Therapy with fluoroquinolones  C: Compared to antibiotic therapy with amoxicillin-clavulanic acid  O1: Is it equally effective in reducing the severity and/or duration of symptoms?  O2: Does it modify the risk of recurrences?  O3: Does it modify the risk of suppurative complications (orbital cellulitis, brain abscess, other)?  O4: Does it modify the risk of non-suppurative complications (cavernous sinus thrombosis, other)?  O5: Does it modify the risk of side effects? |
| --- | --- |

| **Systematic review** | **Population and Purpose of the SR** | **Results** | **Conclusion** |
| --- | --- | --- | --- |
| Head K. et al. Systemic and topical antibiotics for chronic rhinosinusitis. | **Population:** Adults aged 20 to 70 years, plus a single pediatric study with 79 children aged 2-12 years.  **Antibiotic Therapy:** 3 months of therapy with macrolides or tetracyclines.  **Comparisons:**   - Antibiotic vs. placebo - Antibiotic + intranasal saline vs. placebo + saline - Antibiotic vs. nasal steroid - Antibiotic vs. oral steroid   **Outcome:** Assessment using the SNOT-20 (Sinus Nasal Outcome Test-20). | **Antibiotic vs. placebo:** In 3 studies, there is evidence of a reduction in SNOT-20 scores in the treated group at the end of the treatment, but this evidence is not confirmed 3 months after the end of therapy.  **Antibiotic + intranasal saline vs. placebo + intranasal saline:** No difference between antibiotic and placebo.  **Antibiotic vs. nasal steroid:** Uncertain evidence.  **Antibiotic vs. oral steroid:** Uncertain evidence. | The evidence comes from 5 studies, including only one pediatric study (Otten et al. 1994 – 79 patients). The results are not stratified by age. The heterogeneity of the antibiotic treatments used is not commented on. |

| **Question 9: What is the duration of systemic antibiotic therapy for a child with chronic sinusitis?** | P In children with chronic sinusitis  I1 antibiotic therapy for 10 days  I2 prolonged antibiotic therapy (>14 days)  C compared to antibiotic therapy for 14 days  O1 is equally effective in reducing the severity and/or duration of symptoms?  O2 changes the risk of recurrence?  O3 changes the risk of suppurative complications (orbital cellulitis, brain abscess, etc.)?  O4 changes the risk of non-suppurative complications (cavernous sinus thrombosis, etc.)?  O5 changes the risk of side effects? |
| --- | --- |

Letteratura insufficiente per rispondere al quesito.

| **Question 10: What is the recommended therapy for a patient with uncomplicated sinusitis who is allergic to penicillin?** | P In children with uncomplicated sinusitis and penicillin allergy  I1 therapy with macrolides  I2 therapy with fluoroquinolones  I3 therapy with cephalosporins  C compared to therapy with amoxicillin  O1 is equally effective in reducing the severity and/or duration of symptoms?  O2 changes the risk of recurrence?  O3 changes the risk of suppurative complications (orbital cellulitis, brain abscess, etc.)?  O4 changes the risk of non-suppurative complications (cavernous sinus thrombosis, etc.)?  O5 changes the risk of side effects? |
| --- | --- |

Insufficient literature to answer the question.

| **Question 11: What antibiotic therapy is recommended for a child with a recurrence of uncomplicated acute sinusitis?** | P In children with recurrent uncomplicated acute sinusitis  I1 antibiotic therapy with cephalosporins  I2 antibiotic therapy with fluoroquinolones  I3 prolonged therapy with amoxicillin-clavulanic acid (for 14 days)  C compared to a new cycle of first-line antibiotic (amoxicillin-clavulanic acid for 10 days)  O1 is equally effective in reducing the severity and/or duration of symptoms?  O2 changes the risk of recurrence?  O3 changes the risk of suppurative complications (orbital cellulitis, brain abscess, etc.)?  O4 changes the risk of non-suppurative complications (cavernous sinus thrombosis, etc.)?  O5 changes the risk of side effects? |
| --- | --- |

Insufficient literature to answer the question.

| **Question 12: Is antibiotic prophylaxis recommended for children with recurrent sinusitis?** | P In children with recurrent sinusitis  I antibiotic prophylaxis  C compared to no antibiotic therapy  O1 is it more effective in reducing the severity and/or duration of symptoms during recurrences?  O2 does it change the risk of recurrence?  O3 does it change the risk of suppurative complications (orbital cellulitis, brain abscess, etc.)?  O4 does it change the risk of non-suppurative complications (cavernous sinus thrombosis, etc.)?  O5 does it change the risk of side effects? |
| --- | --- |

| **Study**  **(Author, Year)** | **Study Design** | **Population (Number, Country, Setting, Condition)** | **Intervention/Exposure** | **Primary Outcome** | **Effect Measures** | **Secondary Outcomes** | **Follow-up** | **Results** | **Funding** |
| --- | --- | --- | --- | --- | --- | --- | --- | --- | --- |
| Veskitkul J, 2017 | RCT | 40 children (ages 5-15) with recurrent acute rhinosinusitis (RARS) defined as 3 episodes of AR in 6 months or 4 in 12 months, each lasting < 30 days and separated by intervals of at least 10 days during which the patient is asymptomatic. Thailand.  Assessment criteria: immunocompetence (measurement of IgA, IgG, IgM, IgG subclasses, antibody response to pneumococcal vaccination), allergy (skin-prick test with a panel of prevalent local aeroallergens) | Active group: Azithromycin (5 mg/kg) for 3 days a week on non-consecutive days, for 12 months  Control group: Oral placebo identical in physical characteristics | Number of episodes of recurrent acute rhinosinusitis (RARS) in 12 months | Mann- Whitney U test and Wilcoxon signed-rank test | Measurement of the Visual Analog Scale (VAS) and the Complementary Medication Usage Score (AMS) | 12 months | **Active group:** 20 patients **Control group:** 20 patients All completed the follow-up.  The number of episodes in the active group was significantly reduced after 12 months (median, 0.5; range, 0-6.0) compared to baseline (median, 5.0; range, 4.0-7.0) (difference, 4.0; 95% CI, 5.0 to 3.0; P < .001); in the control group, there was no statistically significant reduction (median at baseline, 4.0, and range, 4.0-6.0; median at trial completion, 4.0, and range, 0-6.0) (difference, 0.0; 95% CI, 2.0 to 0.0; P = .09). The reduction from baseline in the active group was statistically significant compared to the control group (P < .001).  Patients with a reduction in the number of episodes >50% were defined as “responders.” In the active group, 85% (17/20) were responders, while in the control group, 25% (5/20) were responders.  VAS and AMS scores were significantly reduced in the active group compared to the control group. | None |
