# Supplementary material for: Treatment of sinusitis in children: an Italian intersociety consensus (SIPPS-SIP-SITIP-FIMP-SIAIP-SIMRI-SIM-FIMMG)
Source: Ital J Pediatr. 2025 Mar 26;51:102. doi: 10.1186/s13052-025-01868-1 (PMC11948864; doi:10.1186/s13052-025-01868-1)
Supplement: Supplementary file 4 — Supplementary Material 4 [file 13052_2025_1868_MOESM4_ESM.docx]

| \|  \|  \|  \|  \|  \|  \| \| --- \| --- \| --- \| --- \| --- \| --- \| \|  \| \| \| \| \| \| \|  \|  \|  \| \| **Systematic reviews evaluation** \| \| \| \| \| \| \|  \|  \|  \| \|  \| \| \| \| \| \| \|  \|  \|  \| |  |  |  |  |
| --- | --- | --- | --- | --- | --- | --- | --- | --- | --- | --- | --- | --- | --- | --- | --- | --- | --- | --- | --- | --- | --- | --- | --- | --- | --- | --- | --- | --- | --- | --- | --- | --- | --- | --- | --- | --- | --- | --- | --- | --- |
| **AMSTAR 2** | **Smith MJ, 2013** | **Axiotakis Jr, 2022** | **Cronin MJ, 2013** | **Head K, 2016** |
| **1. Did the research questions and inclusion criteria for the review include the PICO components? (Yes No)** | Yes | Yes | Yes | Yes |
| **2. Did the report of the systematic review contain an explicit statement that the methods of the review were established prior to conducting the review, and did the report justify any significant deviations from the protocol? (Yes / Partially Yes / No)** | Partially yes | Partially yes | Partially yes | Yes |
| **3. Did the authors of the review justify their selection of study designs to be included in the review? (Yes No)** | Yes | Yes | Yes | Yes |
| **4.Did the authors of the review use a comprehensive literature search strategy? (Yes / Partially Yes / No)** | Yes | Yes | Partially yes | Yes |
| **5.Did the authors of the review perform study selection in duplicate? (Yes No)** | No | Yes | Yes | Yes |
| **6.Did the authors of the review perform data extraction in duplicate? (Yes No)** | No | Yes | Yes | Yes |
| **7.Did the authors of the review provide a list of excluded studies and justify the exclusions? (Yes / Partially Yes / No)** | Yes | Partially yes | Partially yes | Yes |
| **8.Did the authors describe the included studies in sufficient detail? (Yes / Partially Yes / No)** | Yes | Yes | Yes | Yes |
| **9.Did the authors use a satisfactory technique to assess the risk of bias (RoB) in individual studies included in the review? (Yes / Partially Yes / No / Includes only NRSI-RCT)** | Include solo NRSI-RCT | Yes | Yes | Yes |
| **10.Did the authors report the sources of funding for the studies included in the review? (Yes No)** | No | No | No | Yes |
| **11.If a meta-analysis was conducted, did the authors use appropriate methods for the statistical aggregation of results? (Yes / No / No meta-analysis conducted)** | No meta-analysis | Yes | Yes | No meta-analysis |
| **12.If a meta-analysis was conducted, did the authors assess the potential impact of RoB in individual studies on the meta-analysis results or other evidence syntheses? (Yes / No / No meta-analysis conducted)** | No meta-analysis | Yes | Yes | No meta-analysis |
| **13.Did the authors take RoB in individual studies into account when interpreting/discussing the results of the review? (Yes No)** | No | Yes | Yes | Yes |
| **14.Did the authors provide a satisfactory explanation and discussion of any heterogeneity observed in the review results? (Yes No)** | Yes | Yes | Yes | Yes |
| **15.If they conducted a quantitative synthesis, did the authors perform an adequate investigation of publication bias (bias from small studies) and discuss its likely impact on the review results? (Yes / No / No meta-analysis conducted)** | No meta-analysis | No | No | No meta-analysis |
| **16.Did the authors report potential sources of conflict of interest, including any funding received for conducting the review? (Yes No)** | No | Yes | Yes | Yes |
| **OVERALL EVALUATION** | **Low** | **Moderate** | **Moderate** | **High quality** |
| *** Presence of 1 critical item and 2 non-critical items failed (nos. 3, 15, and 16)** |  |  |  |  |

| **Excluded systematic reviews with justification** | |
| --- | --- |
| **SR excluded** | **Reason for exclusion** |
| Clement WA, Sooby P, Doherty C, Qayyum N, Irwin G. Acute isolated sphenoid sinusitis in children: A case series and systematic review of the literature. Int J Pediatr Otorhinolaryngol. 2021;140 | Irrelevant topic |
| Alves Galvão M, Rocha Crispino Santos M, Alves da Cunha A. Antibiotics for preventing suppurative complications from undifferentiated acute respiratory infections in children under five years of age. Cochrane Database of Systematic Reviews. 2016;(2). | Irrelevant topic |
| Dawson-Hahn EE, Mickan S, Onakpoya I, et al. Short-course versus long-course oral antibiotic treatment for infections treated in outpatient settings: a review of systematic reviews. Fam Pract. 2017;34(5):511-519. | Adult |

| **Study Evaluation** | | | | | | | | | |  |
| --- | --- | --- | --- | --- | --- | --- | --- | --- | --- | --- |
|  | **Newcastle Quality Assessment Scale** | | |  |  |  |  |  | |  |
|  | **Selection** |  |  |  | **Comparabilità** | **Outcome** |  | |  |  |
| **Study** | **Representativeness of the exposed cohort** | **Selection of the unexposed cohort** | **Verification of exposure** | **Demonstration that the outcome of interest is not present at the start of the study** | **Comparability of cohorts based on design or analysis** | **Outcome evaluation** | **Was the follow-up long enough for the outcome to occur?** | | **Adequacy of cohort follow-up** | **Total** |
| **Cushen et al. 2019** | 1a | 1a | 1a | 1a | 1a (1 star out of 2: Only some important confounding factors were considered, but additional ones were not.) | 1b | 1a | | 1a | 8/9 |
|  |  |  |  |  |  |  |  | |  |  |
|  |  |  |  |  |  |  |  | |  |  |
|  |  |  |  |  |  |  |  | |  |  |

**RCT (Nordic Cochrane Centre, The Cochrane Collaboration. Review Manager 5 (RevMan 5). Version 5.3. Copenhagen: Nordic Cochrane Centre, The Cochrane Collaboration, 2014)**

**Risk of bias graph: review authors' judgements about each risk of bias item presented as percentages across all included studies**

**
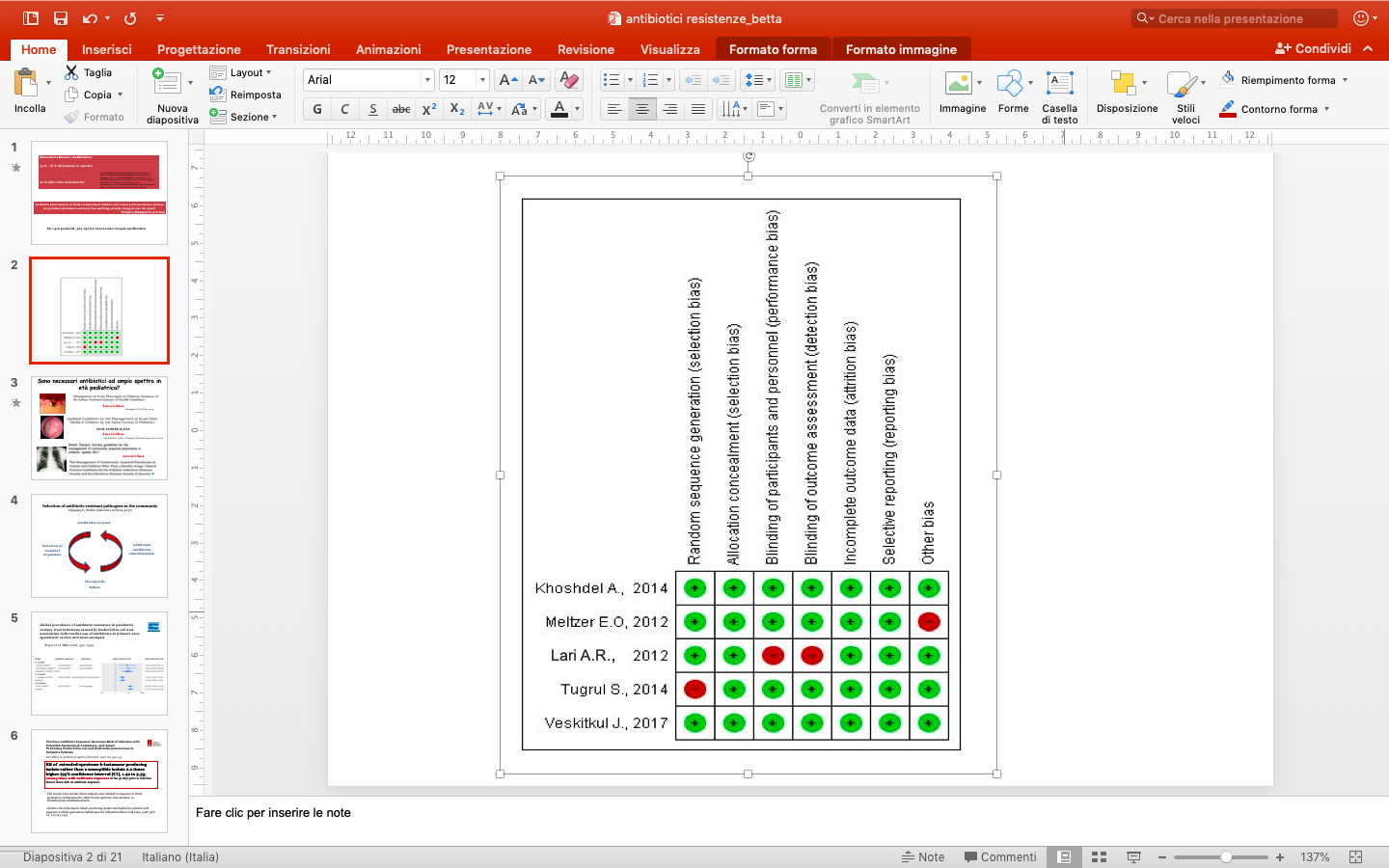
**

**
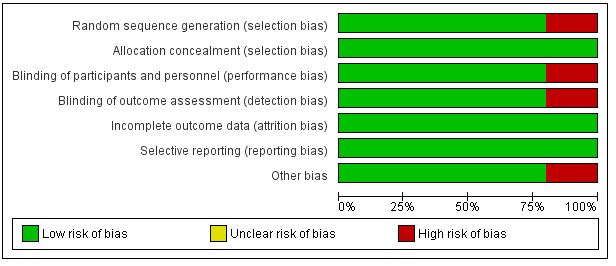
**

**Excluded studies with justification**

|  | |
| --- | --- |
| **Excluded studies** | **Reason for exclusion** |
| Sabino HA, Valera FC, Aragon DC, et al. Amoxicillin-clavulanate for patients with acute exacerbation of chronic rhinosinusitis: a prospective, double-blinded, placebo-controlled trial. Int Forum Allergy Rhinol. 2017;7(2):135-142. | Adult |
| Koyama T, Hagiya H, Teratani Y, et al. Antibiotic prescriptions for Japanese outpatients with acute respiratory tract infections (2013–2015): A retrospective Observational Study. J Infect Chemother. 2020;26(7):660-666. | Irrilevant topic |
| Improving Antibiotic Prescribing for Pediatric Respiratory Infections by Family Physicians With Peer Comparison. https://clinicaltrials.gov/show/NCT04588376. Published online 2020. | Irrilevant topic |
| Kronman MP, Zhou C, Mangione-Smith R. Bacterial prevalence and antimicrobial prescribing trends for acute respiratory tract infections. Pediatrics. 2014;134(4):e956-e965. | Irrilevant topic |
| Dekker AR, Verheij TJ, van der Velden AW. Inappropriate antibiotic prescription for respiratory tract indications: most prominent in adult patients. Fam Pract. 2015;32(4):401-407. | Irrilevant topic |
| Speakman J, Srinivasand S, Taggart L, et al. Index of suspicion. Pediatr Rev. 2013;34(10):465-473. | Irrilevant topic |
| Zhao SR, Griffin MR, Patterson BL, et al. Risk Factors for Outpatient Use of Antibiotics in Children with Acute Respiratory Illnesses. South Med J. 2017;110(3):172-180. | Irrilevant topic |
| Lopatin AS, Ivanchenko OA, Soshnikov SS, Mullol J. Cyclamen europaeum improves the effect of oral antibiotics on exacerbations and recurrences of chronic rhinosinusitis: a real-life observational study (CHRONOS). Acta Otorhinolaryngol Ital. 2018;38(2):115-123. | Irrilevant topic |
| Mösges R, Desrosiers M, Arvis P, Heldner S. Characterisation of patients receiving moxifloxacin for acute bacterial rhinosinusitis in clinical practice: results from an international, observational cohort study. PLoS One. 2013;8(4):e61927. | Adult  Post-marketing study |
| R Deepa, R Jyothi, H P Pundarikaksha, B Jagannath. A study on the drug prescribing pattern in para-nasal sinusitis at a tertiary care hospital. [Natl J Physiol Pharm Pharmacol](https://www.bibliomed.org/?jtt=2320-4672). 2014; 4(3): 182-186 | Irrilevant topic |
| Seresirikachorn K, Chetthanon T, Suwansirisuk T, et al. Low-dose macrolides for treating pediatric rhinosinusitis: A retrospective study and literature review. SAGE Open Med. 2020;8: Published 2020 Jun 30. | Irrilevant topic |
| Zhao Q, Yu L, Jin P, Ma W, Duan S, Luo H. A comprehensive investigation of the demographics, treatments, comorbidities, and disease burden of chronic rhinosinusitis with nasal polyposis patients: a descriptive analysis. Ann Transl Med. 2022 Feb;10(3):150. | Irrilevant topic |
| Veskitkul J, Vichyanond P, Pacharn P, Visitsunthorn N, Jirapongsananuruk O. Clinical characteristics of recurrent acute rhinosinusitis in children. Asian Pac J Allergy Immunol. 2015 Dec;33(4):276-80. | Irrilevant topic |
| Poachanukoon O, Tangsathapornpong A, Tanuchit S. A Comparison of Cefditoren Pivoxil 8-12 mg/kg/day and Cefditoren Pivoxil 16-20 mg/kg/day in Treatment of Children With Acute Presumed Bacterial Rhinosinusitis: A Prospective, Randomized, Investigator-Blinded, Parallel-Group Study. Clin Exp Otorhinolaryngol. 2015 Jun;8(2):129-35 | Irrilevant topic |
