# Supplementary material for: Treatment of sinusitis in children: an Italian intersociety consensus (SIPPS-SIP-SITIP-FIMP-SIAIP-SIMRI-SIM-FIMMG)
Source: Ital J Pediatr. 2025 Mar 26;51:102. doi: 10.1186/s13052-025-01868-1 (PMC11948864; doi:10.1186/s13052-025-01868-1)
Supplement: Supplementary file 5 — Supplementary Material 5 [file 13052_2025_1868_MOESM5_ESM.docx]

**Second line**: amoxicillin-clavulanate or cephalosporin (e.g., cefixime, cefpodoxime, cefuroxime) and clindamycin

clinical worsening (or no improvement after 48-72 hours)

**Amoxicillin 90 mg/kg/die in 3 daily doses** (category 1)

**Amoxicillin-clavulanate 90 mg/kg/die** (of amoxicillin) **in 3 daily doses** (category 2)

**ALLERGY:** low risk of severe allergic reaction 🡪 third-generation cephalosporin; high risk 🡪 quinolone

**for 10 days**

no topical antibiotic

2 episodes in <30 days, with at least 10 days of wellness

at least 3 episodes in 6 months or 4 in 12 months (each lasting less than 30 days)

>12W/90 days

**ACUTE SINUSITIS**

**DIAGNOSIS 🡪 CLINICAL**

**IMAGING**

- Not recommended in uncomplicated acute sinusitis
- Persistent, severe, recurrent, or therapy-resistant sinusitis: direct CT is the gold standard.
- Chronic sinusitis: direct CT is not superior to clinical history and examination; MRI can be useful for studying the mucosa.
- In cases of therapy-resistant sinusitis, CT with coronal, sagittal, and axial sections is useful and can provide information about complete/partial sinus opacification, the presence of air-fluid levels, mucosal thickness, and any anatomical alterations requiring surgical intervention

**Not possible** to give any specific recommendation regarding prophylaxis

**RECURRENT ACUTE SINUSITIS**

Amoxicillin in previous episode 🡪 amoxicillin-clavulanate

Poor compliance with amoxicillin-clavulanate in previous episode 🡪 repeat the therapeutic cycle with the same antibiotic

Good compliance with amoxicillin-clavulanate in previous episode 🡪second-line antibiotic therapy

Specialist consultation

**RECURRENCE**

Antibiotics not recommended

Before perform invasive diagnostic interventions 🡪 prescribe a course of antibiotic therapy if not previously administered (amoxicillin-clavulanic)

Before undertaking invasive diagnostic interventions 🡪 specialist consultation

1. Sudden onset of ≥ 2 symptoms among nasal obstruction/congestion, nasal discharge, daytime or night-time cough lasting at least 10 days without improvement for a total duration of <12 weeks/90 days (excluding allergic subjects);
2. Persistent malaise, worsening or new onset of nasal discharge, daytime cough or fever after initial improvement;
3. Severe onset with fever (temperature ≥39°C) and purulent nasal discharge for at least 3 consecutive days.

**CHRONIC SINUSITIS**

**Amoxicillin-clavulanate 90 mg/kg/die** (of amoxicillin) **in 3 daily doses**

for 10 days

4-12W/30-90 days

**SUB-ACUTE**

**SINUSITIS**

<12W/90 days
